# Electrocatalytic CO_2_ Reduction on CuO_*x*_ Nanocubes: Tracking the Evolution of Chemical State, Geometric Structure, and Catalytic Selectivity using Operando Spectroscopy

**DOI:** 10.1002/anie.202007136

**Published:** 2020-08-13

**Authors:** Tim Möller, Fabian Scholten, Trung Ngo Thanh, Ilya Sinev, Janis Timoshenko, Xingli Wang, Zarko Jovanov, Manuel Gliech, Beatriz Roldan Cuenya, Ana Sofia Varela, Peter Strasser

**Affiliations:** ^1^ The Electrochemical Energy, Catalysis, and Materials Science Laboratory Department of Chemistry Chemical Engineering Division Technical University Berlin Berlin Germany; ^2^ Department of Interface Science Fritz-Haber Institute of the Max Planck Society 14195 Berlin Germany; ^3^ Department of Physics Ruhr-University Bochum 44780 Bochum Germany; ^4^ Institute of Chemistry National Autonomous University of Mexico Mexico City Mexico

**Keywords:** CO_2_ reduction, copper, electrocatalysis, nanocubes, operando spectroscopy

## Abstract

The direct electrochemical conversion of carbon dioxide (CO_2_) into multi‐carbon (C_2+_) products still faces fundamental and technological challenges. While facet‐controlled and oxide‐derived Cu materials have been touted as promising catalysts, their stability has remained problematic and poorly understood. Herein we uncover changes in the chemical and morphological state of supported and unsupported Cu_2_O nanocubes during operation in low‐current H‐Cells and in high‐current gas diffusion electrodes (GDEs) using neutral pH buffer conditions. While unsupported nanocubes achieved a sustained C_2+_ Faradaic efficiency of around 60 % for 40 h, the dispersion on a carbon support sharply shifted the selectivity pattern towards C_1_ products. Operando XAS and time‐resolved electron microscopy revealed the degradation of the cubic shape and, in the presence of a carbon support, the formation of small Cu‐seeds during the surprisingly slow reduction of bulk Cu_2_O. The initially (100)‐rich facet structure has presumably no controlling role on the catalytic selectivity, whereas the oxide‐derived generation of under‐coordinated lattice defects, can support the high C_2+_ product yields.

## Graphical abstract

Cu_2_O cubes of nanometer‐sized dimensions allow the chemical and structural factors that control the selectivity of the CO_2_ reduction reaction (CO_2_RR) to be traced. The Faradaic product efficiencies over time can be linked to changes in the chemical state at the surface and bulk and in the catalyst morphology for supported and unsupported nanocubes (S‐NC, U‐NC).

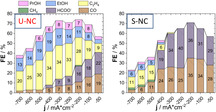

## Introduction

A society fueled by intermittent electricity from wind and solar power plants invariably requires electrochemical technologies to store recurring electricity supply surpluses.^1^ The direct CO_2_ electrochemical reduction reaction (CO_2_RR) has emerged as one promising technology to use electricity to convert CO_2_ into carbon‐based chemicals or fuels, thereby closing the anthropogenic energy carbon cycle.^2^

The product distribution of the CO_2_RR depends sensitively on the chemical nature of the catalyst, in particular Cu has been intensively studied and is arguably one of the most interesting materials for the CO_2_RR due its capability of facilitating the direct reduction of CO_2_ into C_2+_ products.^3^, ^4^ However, the required kinetic overpotentials, issues of selectivity and stability still largely limit commercial interest. To date, most Cu‐based catalysts employed in CO_2_RR revealed a complex temporal variation of the faradaic efficiency. The origin of these efficiency variations remains, however, poorly explored. Some of these changes can be assigned to the deposition of electrolyte contaminations, while others arise from the complex structural and chemical transformation of the catalysts.^5^

In recent years, much scientific work has been focused on understanding the fundamental factors that control the catalytic activity of Cu. Different studies have repeatedly shown that the selectivity between C_2_H_4_/CH_4_ is strongly dependent on the surface structure and composition of the catalyst, as well as on the reaction electrolyte.^5^, ^6^ It has been established, that while ethylene is the predominant hydrocarbon in alkaline pH, the production of CH_4_ is favored in acidic conditions, due to a difference in the mechanistic protonation pathway.^7^ In the case of nanoparticles, both the size and the interparticle distance showed an effect on the reaction activity and selectivity.6d, 8 Work on polycrystalline Cu implied that the C_2_H_4_ to CH_4_ ratio is strongly dependent on the surface pretreatment. In particular, oxide derived Cu (OD‐Cu) exhibited a clearly altered catalytic performance from that of metallic Cu, suppressing CH_4_ selectivity and lowering the onset potential for CO and C_2_H_4_ formation.2d, 6a, 6j, 9 Despite the promising results obtained on OD‐Cu, the molecular origin of its unique selectivity has remained controversial. A number of studies worked on the hypothesis that the outstanding catalytic performance of OD‐Cu may origin in an altered binding strength of reactive intermediates. Here, different causes such as grain boundaries,^10^ the presence of remaining Cu^I[2d, 11]^ and of subsurface oxygen were suggested.6g, 9b, 12

On a device site, Gas Diffusion Electrodes (GDEs) have proved indispensable for integration of catalytic reactions in larger‐scale electrolyzer devices, as shown for the case of CO_2_ to CO with GDEs based on Ag catalysts, pioneered by the Kenis group.^13^ To date, Cu catalyst‐based GDE studies have largely included electrodeposited Cu films for the production of hydrocarbons and oxygenates, yet their yields, reactivity and performance stability remained low.^14^ Later, more efficient Cu catalyst‐based GDEs operated in electrolyzer cells were reported, at the price of using highly alkaline, and therefore quite impractical electrolytes or hazardous pure CO input feeds.^15^

In this contribution, we explore structure and composition‐selectivity relations of cubic, Cu_2_O nanoparticles of about 35 nm edge‐length. The nanocubes were initially tested in a two‐chamber H‐Cell as a carbon‐supported and unsupported powder catalyst. We observed a clear effect of the carbon support, steering the selectivity away from C_2+_ of the unsupported nanocubes towards C_1_ products. We correlated this difference to an altered morphological evolution of the nanocubes by spatial isolation of the particles on the conductive support. Furthermore, we monitored the surprisingly slow and incomplete electrochemical reduction of the unsupported Cu_2_O particles on the molecular scale. Our *operando* XAS allowed us to trace the electrochemical reduction of the initially cubic Cu_2_O into predominantly metallic particles of ill‐defined morphology, showing an abundance of Cu lattice defects, which are often associated with catalytic sites of extraordinary activity. In this, we succeed at presenting the formation of a defective Cu structure by electrochemical reduction of an oxidized precursor in real time.

Furthermore, deposition of the catalysts on a Gas Diffusion Electrode allowed for tests at industrially relevant current densities of 50 to 700 mA cm^−2^ in an electrolyzer flow cell. We were able demonstrate a high and stable faradaic efficiency of unsupported Cu_2_O nanocubes toward C_2+_ products in an electrolyzer set up at neutral pH. Carbon‐supported Cu_2_O nanocubes, on the other hand, undergo distinctly different structural dynamics in electrolyzers. Over the course of 40 h, they first form very small Cu seeds that grow, sinter, and eventually resemble the unsupported Cu particles in structure and efficiency.

## Results and Discussion

### Unsupported and carbon‐supported cubic Cu_2_O nanocatalysts

**Figure 1:**
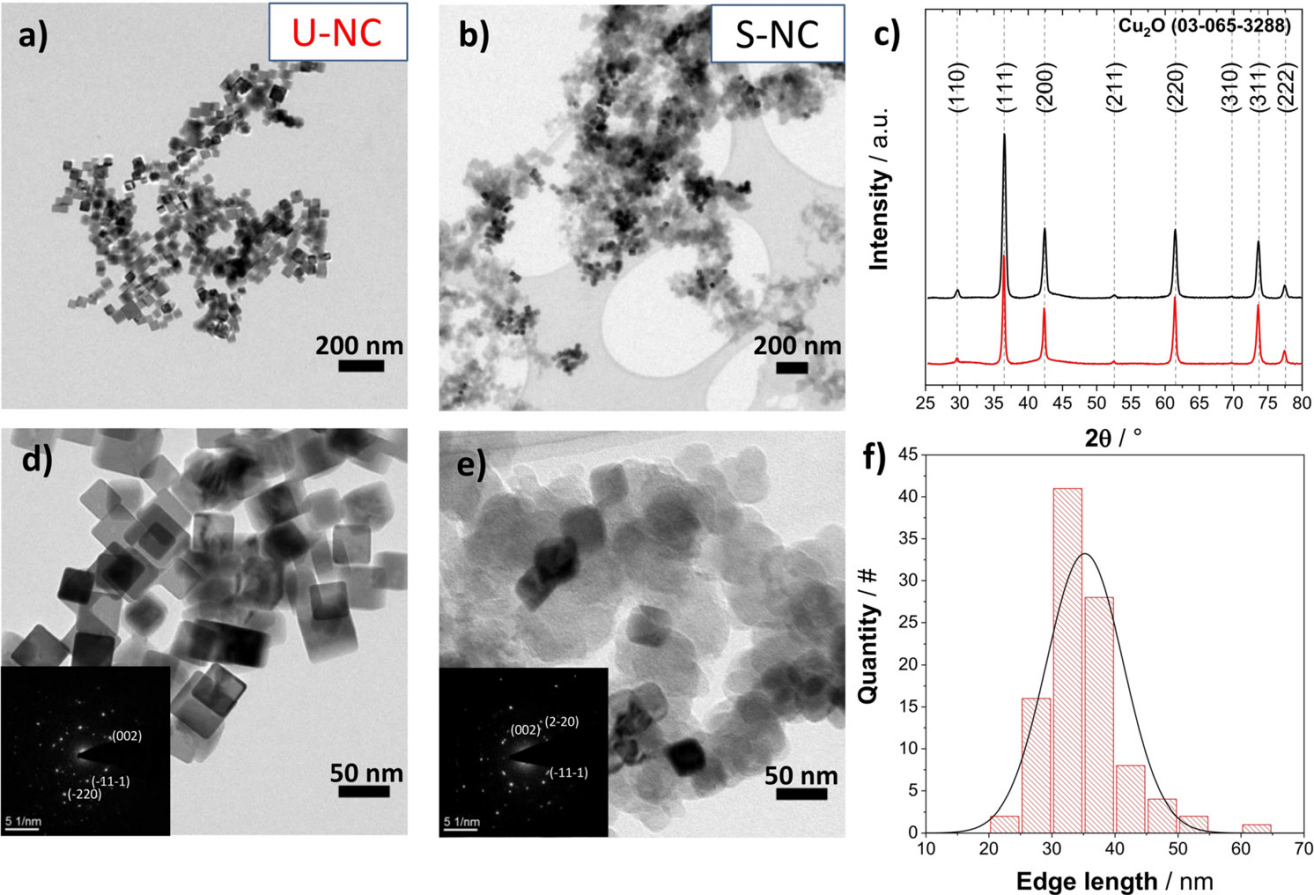
Transmission electron microscopy (TEM) images of a),d) the unsupported Cu_2_O nanocubes, U‐NC, and b),e) the carbon‐supported Cu_2_O nanocubes (23 wt %), S‐NC. Insets in (d,e) show selected area electron diffraction (SAED) patterns of the respective material. c) X‐ray diffraction (XRD) patterns of both catalysts and f) particle size histogram derived from TEM images of the U‐NC.

The unsupported Cu_2_O nanocubes will be referred to as “U‐NC”, while the supported nanocubes at a loading of 23 weight% will be denoted as “S‐NC”. Figure 1 a–e and Figure S1b show the local TEM‐based microstructural morphologies of the as‐prepared unsupported and supported nanocatalysts, while the insets in Figure 1 d,e display the selected area of the electron diffraction patterns and Figure 1 c the X‐ray diffraction patterns of the crystalline phases, respectively. Figure 1 d,e and Figure S1b confirm the targeted cubic morphology of the Cu‐based NPs with an edge length of 35±6 nm (see histogram in Figure 1 f), which showed no apparent change once supported on the carbon. The crystal phase analysis in Figure 1 d,e,c revealed a single Cu_2_O phase for both S‐NCs and the U‐NCs, confirming the stability of the crystalline cubic Cu_2_O phase during the supporting procedure.

### Electrochemical CO_2_RR activity and stability in a liquid‐electrolyte H‐Cell configuration

Subsequently, both CO_2_RR electrocatalysts, S‐NC and U‐NC, were tested in a two‐compartment H‐Cell at constant applied electrode potentials in aqueous 0.1 m KHCO_3_ buffer electrolyte. While the favorable faradaic ethylene efficiencies of cubic‐shaped Cu‐based nanoparticles have been documented in previous works,^16^ catalytic support effects have been proven important but remain less studied.^5^ Therefore, we place emphasis on the comparison between unsupported nanocubes, U‐NC (red color code and symbols), and the supported ones, S‐NC (black code and symbols).

**Figure 2:**
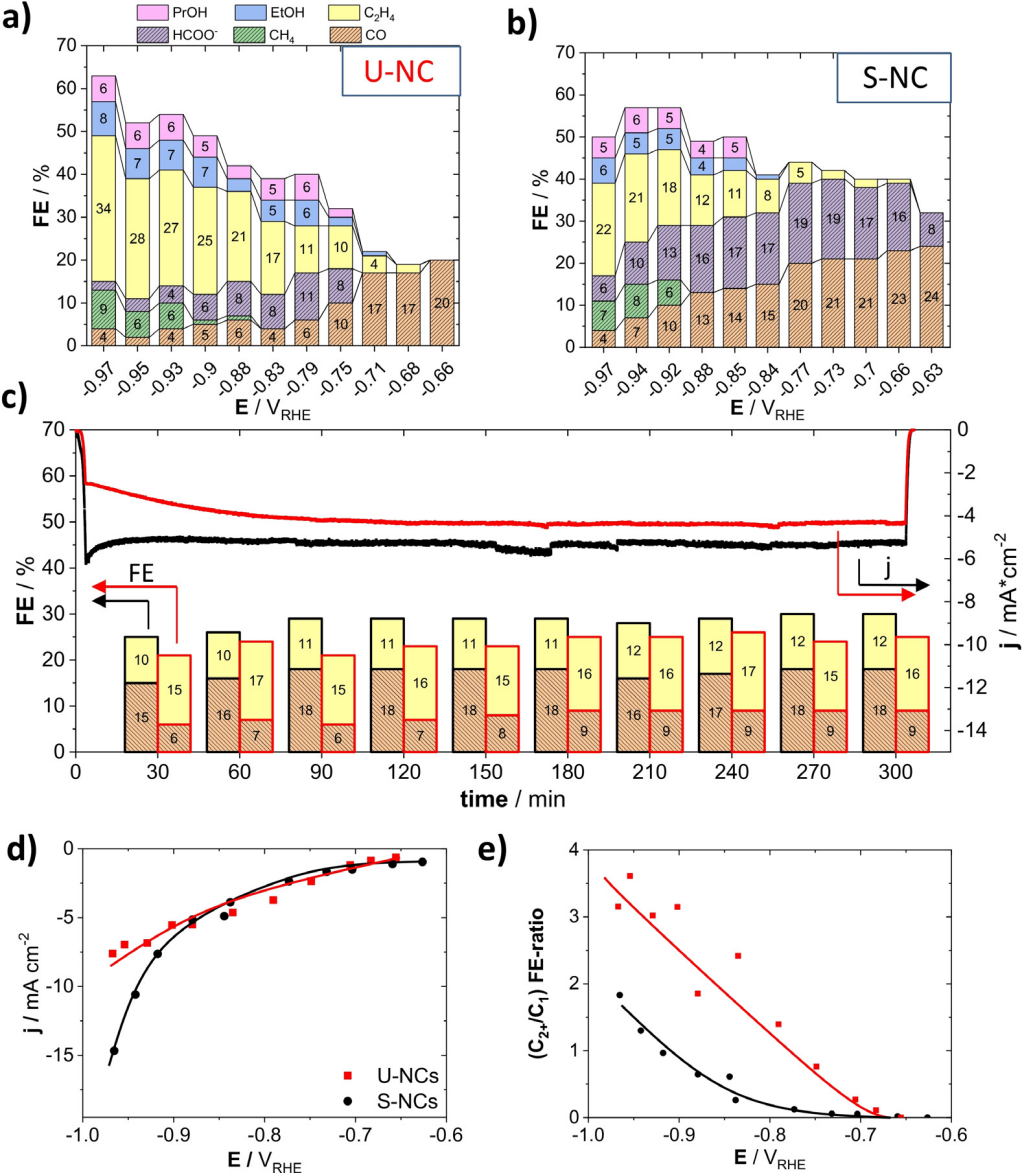
a),b) Faradaic product efficiencies (FEs) as a function of the applied electrode potential after one hour of reaction time for a) the unsupported Cu nanocubes, U‐NC and b) the carbon‐supported Cu nanocubes (23 wt %), S‐NC. Color coded bars denote products as given in the legend. c) Chronoamperometric efficiency stability at a constant applied electrode potential of −0.86 V_RHE_ for the U‐NC and the S‐NC catalysts. Colors as in (a). d) The electrochemical CO_2_ reduction polarization curves (geometric current‐density vs. IR‐corrected applied electrode potential) and e) (C_2+_/C_1_) FE ratios versus IR corrected applied electrode potential. Lines to guide the eye.

**Catalytic CO_2_RR Activity**: The S‐NC showed a clearly higher geometric current density compared to the U‐NC at more cathodic potentials than −0.9 V_RHE_ (Figure 2 d). Comparison to a supported sample of higher particle loading (44 wt %) showed a similar enhancement in activity, agreeing with the notion of a higher availability of active Cu sites by dispersion on the support, see Figure S5. To exclude effects of the substrate (glassy carbon plate) and support (Vulcan carbon) reference measurements were performed, which showed only minor catalytic activity of the two, mainly attributable to HER (Figure S4). We further performed measurements of lead under potential deposition (Pb‐UPD) in order to quantify this increase in accessible surface, which we estimated to be in the order of 3 times for the S‐NC in comparison to the U‐NC based on the respective Pb‐monolayer stripping charge (Figure S3).

**Catalytic CO_2_RR Selectivity**: Figure 2 a,b shows the Faradaic efficiency (FE) values of all CO_2_RR products for the U‐NC and S‐NC as function of the applied IR‐corrected potential. At potentials of −0.65 V_RHE_, both catalysts exhibited high CO FEs, early onset potentials of ethylene combined with a suppression of methane formation. At −0.97 V_RHE_, the U‐NCs displayed high ethylene selectivity, given the neutral pH conditions, exceeding 30 % FE. The high C_2+_ product yield is consistent with previous reports on CO_2_ electroreduction on cubic‐shaped Cu NPs in H‐Cell, and is considered to constitute the key advantage of Cu(100) facet‐rich nanocatalysts over electropolished Cu foils.16b, 17

In addition to the distinct catalytic activity, the dispersion of the Cu nanocubes on the carbon support induced important differences in terms of selectivity: The S‐NC displayed a clearly lower ethylene FE, while the FEs of CO and HCOO^−^ were higher. The HCOO^−^ FE peaked at 19 %, CO at 24 %, while the C_1_ FEs values generally remained at elevated levels. This observation is in good agreement with previous studies.^5^ Again, our supported 44 wt % Cu nanocubes sample is in agreement with the observed FE shift, caused by the support, see Figure S1.

Next, we turn to a comparison of the time‐stability of the FE values of the S‐NC and the U‐NC catalysts at −0.86 V_RHE_, displayed in Figure 2 c. It is evident that both catalysts displayed distinct, yet similarly time‐stable trajectories of the FE values of their major gas products, resulting from CO_2_RR, over at least 5 hours. Tests at other kinetic overpotentials as −0.95 V_RHE_ and −0.66 V_RHE_ showed again constant FEs over 5 hours of testing time, confirming the stability of the systems (see Figure S6).

**Support effects**: The dispersion of Cu_2_O nanocubes on a carbon support altered their catalytic selectivity in a characteristic way: C_2‐3_ products, such as ethylene, ethanol and propanol became suppressed, whereas the production of C_1_ products, such as CO, HCOO^−^ and CH_4_, was favored. This resulted in a clearly smaller ratio of (C_2+_/C_1_) products over the tested potential range (Figure 2 e). Previously, simple physical mixing of Cu NPs with Ketjenblack carbon prior to electrode casting showed similar shifts in faradaic selectivities, and was attributed to a disrupted morphological particle evolution during CO_2_RR from spherical to cubic morphologies.^18^ While that view placed emphasis on dynamic structural changes of Cu particles on carbon surfaces, it neglects the contributing effect of an effectively larger mean interparticle distance on a support, associated with less likely re‐adsorption of reactive intermediates such as CO.6d, 8a Such an effect can be of significance for the S‐NCs, as the dispersion of the Cu nanocubes on the high surface area carbon support, as evidenced by our TEM and Pb‐UPD measurements, results in an effective physical separation of active surface sites of adjacent Cu cubes. In this, we believe that our observation is fundamentally comparable to the work of Wang et al. mentioned above. The reduction of geometric mass loading of spherical CuO_*x*_ particles on planar carbon substrate is similar to the support of Cu_2_O on a porous carbon. In both cases, the spatial density of the particles was reduced in the process, which resulted in a decrease of FE for multi‐carbon products. Furthermore, Grosse et al. recently showed a strong dependence of the experimental CO_2_RR reactivity of electrodeposited, several hundred nanometer‐sized Cu cubes on their underlying substrates, which was chosen as either a Cu foil, or a carbon paper. Their analysis suggested next to a difference in the morphological stability, a difference in chemical stability as well, caused on the substrate.^5^

**Figure 3:**
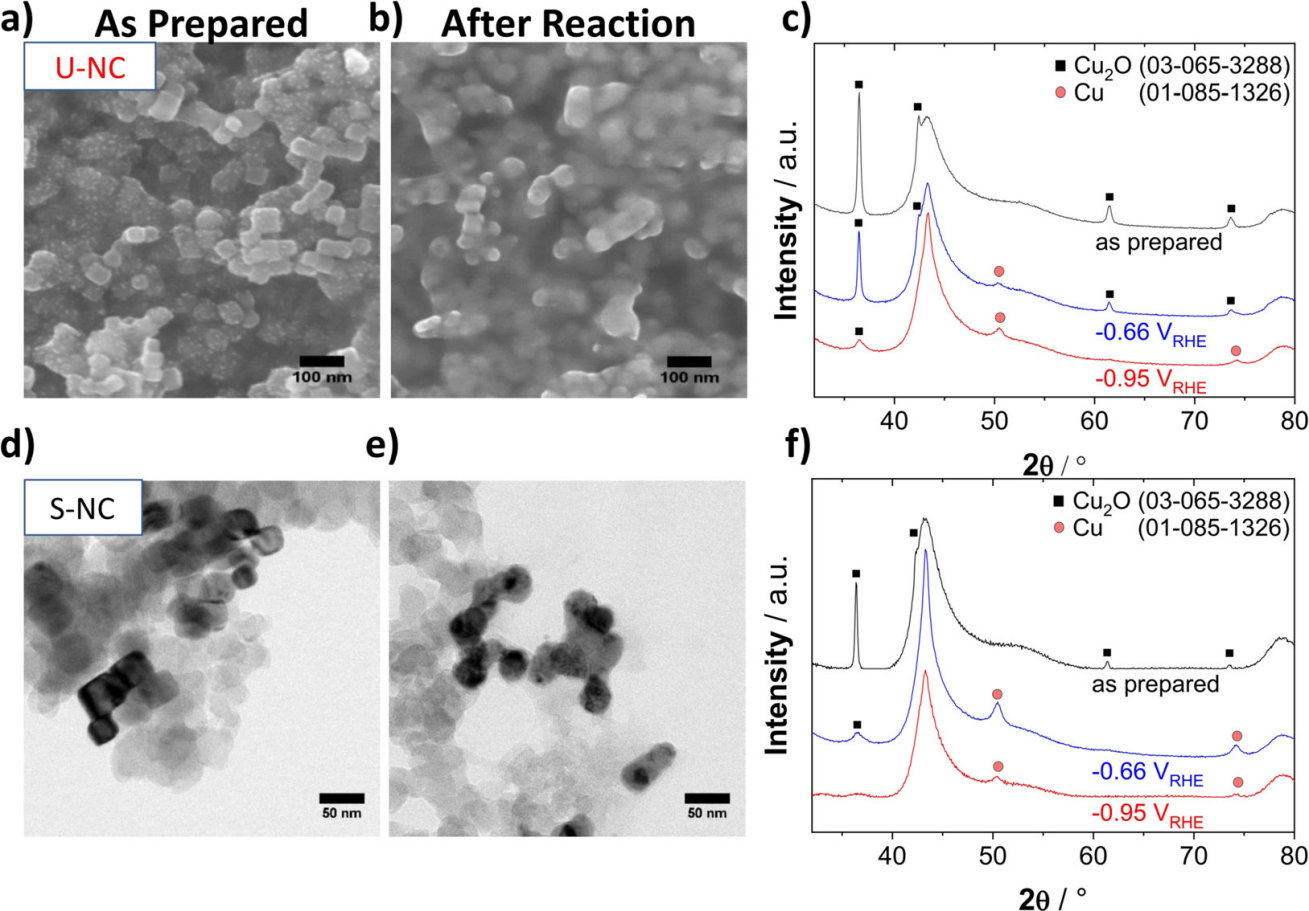
a) SEM images of the unsupported catalyst, U‐NC, as prepared and b) after 1 hour of CO_2_RR at −0.95 V_RHE_. d) TEM images of supported Cu nanocube, S‐NC, (23 wt %) catalyst, as prepared and e) after 1 hour of CO_2_RR testing at −0.95 V_RHE_. c) XRD patterns of the U‐NC catalyst and f) the S‐NC catalysis after initial deposition on a glassy carbon electrode (black), after 1 hour of CO_2_RR at −0.66 V_RHE_ (blue) and after 1 hour at −0.95 V_RHE_ (red). XRD patterns of the blank glassy carbon plate and of the as prepared catalyst powders are displayed in Figure S7 for reference.

**The stability and role of {100} facets**: To further deconvolute the possible origins of the observed catalytic difference, we tracked the morphological and crystal phase evolution of the S‐NC and U‐NC catalysts. Figure 3 a,b and Figure d,e show electron micrographs of both catalysts before and after 60 min of continuous CO_2_ electrolysis at constant potential of −0.95 V_RHE_. The particles appear to have agglomerated and merged, without a defined resulting morphology. Further microscopy of higher resolution clearly shows this strong morphological transition, as seen in Figure S2. From this, we conclude that it is unlikely that the observed stable FE over 5 hours (Figure 2 c, Figure S6) originate from the well‐defined initial cubic morphology. Previous studies on metallic Cu cubes, have attributed their high C_2_ selectivity to the sustained stable presence of {100} facets,16b which are known to catalyze the CO dimerization at low overpotentials.^19^

**Oxide‐derived Cu**: While the mechanistic origins of enhanced dimerization products on {100} facets are well described, the molecular reasons for the improved performance of oxide‐derived Cu (OD‐Cu) surfaces are still controversial. Different factors, such as an increase of the local pH due to large surface roughness compared to a polished metallic Cu foil,9c the presence of grain boundaries after reduction,^20^ remaining oxygen species within the catalyst surface2d, 16c and the formation of undercoordinated Cu sites6b have all been correlated and associated with the high selectivity towards C_2+_ products.

**Origins of sustained high C_2+_ yields**:. Our results suggest that stable C_2+_ FE similar to OD‐Cu bulk catalysts can be obtained on unsupported Cu^I^ oxide nanoparticles as well.20b We hypothesize that factors, such as the detailed geometric nature of the resulting stepped OD‐Cu surfaces, or the chemical state of the Cu atoms at the catalyst surface control the experimental product selectivity during CO_2_RR. Such possible origins have been discussed in recent literature: Mistry et al. showed high ethylene selectivity during the CO_2_RR for an oxygen‐plasma treated copper foil, which partially reduced under reaction conditions, yet STEM‐EDS analyses after the catalytic tests suggested the sustained presence of a small fraction of oxygen atoms and Cu^I^.2d A possible theoretical explanation for this observation was provided by Xiao et al., who suggested that a mixed matrix of metallic and oxidized copper can facilitate the dimerization of adsorbed CO.^21^ Furthermore, the effect of residual sub‐surface oxygen, generated during the reduction of oxidized copper, is discussed in multiple publications and an amorphous metallic Cu layer was suggested as a possible stabilizing site for them.9b, 12a, 12b, 12c, 22 Moreover, the stabilization of Cu^I^ species has also been demonstrated in the presence of Br and I.6j, 12d, 12e

### Post‐reaction, quasi in situ and *operando* catalyst characterization

To gain insight into structural and chemical changes of the surface and bulk of the S‐NCs and U‐NC we investigated first the phase structure of the nanocubes before and after CO_2_RR using grazing incidence X‐ray powder Diffraction (GI‐XRD) (Figure 3 c,f). As expected from the strongly reducing reaction environments, the characteristic metallic Cu(200) Bragg diffraction reflection at 50° indicated the formation of metallic Cu after 60 min of reaction at a potential of −0.65 V_RHE_. Interestingly, the presence of a Cu_2_O(111) facet reflection at 36° remained visible for both catalysts, while the Cu_2_O pattern intensity decreased under more negative potentials (see patterns at −0.95 V_RHE_). This implies a surprising chemical stability of Cu_2_O, contrary to thermodynamic expectations. Nevertheless, we acknowledge the limitations of ex situ XRD to get a proper assessment of the phase‐evolution of the catalyst, especially due to the highly reactive nature of near‐surface metallic copper under ambient conditions. Additionally, changes in nanoparticle orientation can also influence the intensity ratio of observed reflexes in GI‐XRD. This is why we resorted to *quasi* in situ X‐ray Photoelectron Spectroscopy (XPS) to trace the evolution of the chemical state of the surface with *operando* X‐ray Absorption Spectroscopy (XAS) to assess the changes in the bulk phase under reaction conditions.

**Figure 4:**
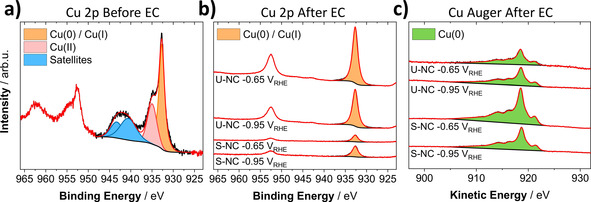
a) XPS Cu 2p of as prepared U‐NC deposited on a glassy carbon electrode. b) Quasi in situ XPS Cu 2p and c) Cu Auger LMM spectra of the U‐NC and S‐NC (23 wt %) catalyst after one hour of CO_2_RR at −0.95 V_RHE_ and −0.65 V_RHE_. Cu AES of as‐prepared catalysts can be found in Figure S8.

The *quasi* in situ XPS measurements (Figure 4 a–c) suggested a complete chemical reduction to metallic Cu of the near surface region after about one hour of reaction at low (−0.65 V_RHE_), as well as more negative (−0.95 V_RHE_) potentials. This change in redox state was comparable for the U‐NC and the S‐NC sample. While our XPS results show a fast reduction of the near surface region, it does not allow for a statement about possible defects introduced during the reduction or the oxidation state of deeper catalyst layers. Here, our analysis by *operando* XAS helps to add further insight into this system.

**Figure 5:**
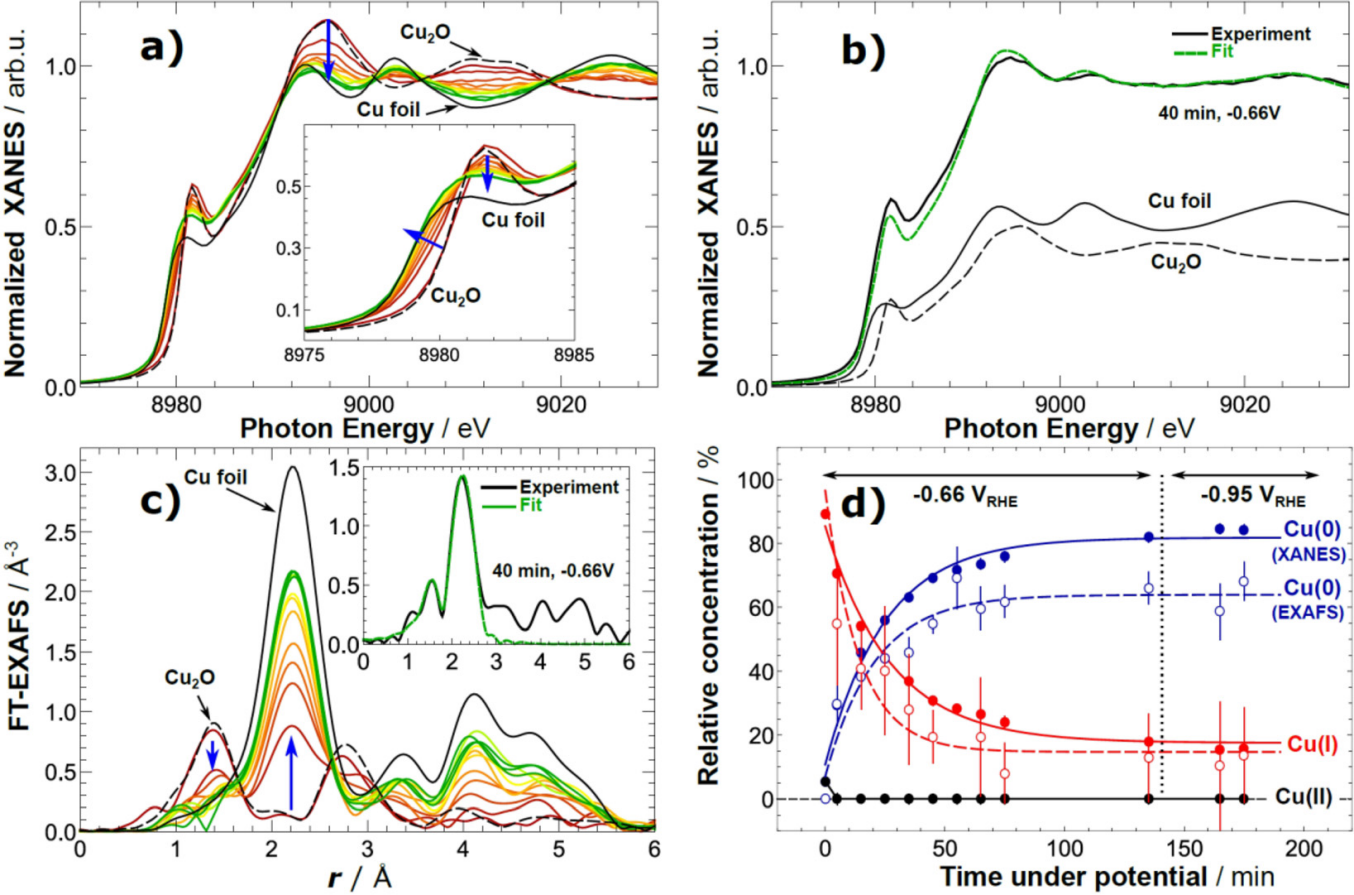
a) Cu K‐edge XANES data of U‐NC sample acquired under operando CO_2_ reduction conditions. b) Representative example of catalyst XANES data fitting with a linear combination of reference spectra (the latter are also shown, scaled by their importance to the analyzed catalyst spectrum). c) Fourier‐transformed (FT) Cu K‐edge EXAFS data of U‐NC sample acquired under operando CO_2_ reduction conditions. Representative example of EXAFS data fitting is shown in the inset. d) Temporal evolution of the chemical composition of the Cu_2_O cubes during CO_2_ electroreduction obtained from the linear combination analysis of XANES data (filled circles) and coordination numbers from EXAFS data fitting (empty circles). Solid and dashed lines are guides for the eye. For additional reference spectra see Figure S9.

The U‐NCs were deployed in an *operando* X‐ray analysis cell and their chemical state and local structure was tracked during the electrochemical reduction of CO_2_. Figure 5 shows *operando* XANES (Figure 5 a) and EXAFS (Figure 5 c) spectra recorded approximately every 10 minutes under chronoamperometric conditions at −0.66 V_RHE_ and subsequently at −0.95 V_RHE_. There were drastic changes taking place in the sample already during the first 10 minutes under the moderate applied potential. While the potential was kept constant, the pre‐edge features became broader, less intense, and the absorption edge shifted to lower energy towards its position in the spectrum of a metallic foil. At the same time, the feature above the edge gradually changed its shape towards a two‐peak feature characteristic of metallic Cu.

A linear combination fitting analysis of the *operando* XANES data, using Cu foil (metallic) and Cu_2_O as basis spectra (Figure 5 b) evidenced a bulk reduction of the Cu_2_O species in the cubes at −0.66 V_RHE_. However, a significant fraction of Cu^I^ species (ca. 20 %) can still be detected even after two hours of reduction in the H‐type cell. Further increase of the potential did not induce any additional changes in the chemical state of copper, and did not result in any reduction of the Cu^I^ species. During the reduction, the Cu local environment as probed by the EXAFS spectra shows a rapid evolution of the metallic Cu‐Cu distance peak at 2.2 Å (uncorrected) accompanied by an abrupt decay in the Cu_2_O‐related peaks (Figure 5 c). For quantitative analysis, we perform EXAFS data fitting (Figure S10 and Table S2). The metallic Cu‐Cu coordination number (CN_Cu‐Cu_) increases from 3.6 after the first 10 minutes of reaction to ca. 8 within 70 minutes and the Cu‐O (CN_Cu‐O_) drops from ca. 2 (as in Cu_2_O) down to 0.3 within the same time (see Table S2 for details). Cu‐Cu CN barely changes when the catalyst was kept at −0.66 V_RHE_ for additional 60 minutes, or when the potential was increased to −0.95 V_RHE_. The findings from the EXAFS data analysis are in agreement with the XANES data. To facilitate the comparison of XANES and EXAFS results, in Figure 5 d we plot Cu‐Cu and Cu‐O coordination numbers, extracted from EXAFS data fitting, divided by respective bulk values (12 and 2, correspondingly) as estimates of concentrations of Cu^0^ and Cu^I^ species. Remarkably, while the coordination number for Cu−O bond, as extracted from EXAFS, is in agreement with concentration of Cu^I^ species, as obtained from XANES analysis, the Cu‐Cu CN is noticeably smaller than expected from XANES analysis, indicating thus the presence of a large amount of undercoordinated Cu sites. Indeed, the final CN was distinctly different from bulk copper and showed no considerable changes even after extended periods of time or after the increase of potential. The presence of undercoordinated atoms gives rise to strong binding‐sites in oxide derived materials, which had been suggested to have an influence on CO_2_RR in studies by Kanan and co‐workers.9a, 10 The stronger binding of reaction intermediates such as CO to defective O_2_‐plasma treated oxidized Cu surfaces was also demonstrated using temperature programmed desorption.12e In a recent theoretical study, Liu et al. supported this idea and suggested that the special performance of recent materials is actually a result of edges and steps introduced to the system,^23^ which has also been proposed in an early work on polycrystalline copper.6b Our results are in line with these studies, and provide new evidence for a formation mechanism of undercoordinated sites by electrochemical reduction of oxidized copper.

Due to the difference in probing depth of the techniques, the combination of XPS and XAS allowed for a quite complementary view on the chemical reduction of the Cu_2_O nanocubes: The catalysts are undergoing a fast reduction at the surface, which is progressing with time towards deeper layers. This could explain the absence of any correlation between the long‐term selectivity and the loss in Cu^I^ species, as the catalyst‐surface became completely reduced after a short reaction time.

### Support effects on catalyst structure and selectivity during long term CO_2_RR in Gas Diffusion Electrodes

To validate the performance at industrial current densities, we deployed the catalysts in commercial GDEs operating in a membrane electrolyzer at neutral pH.

**Figure 6:**
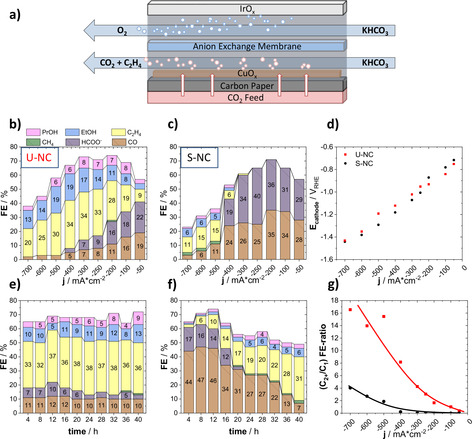
a) Schematic representation of the electrolyzer flow cell. Faradaic product efficiencies as a function of applied geometric current density for b) the U‐NC and c) the S‐NC. e) 40 h stability test at −300 mA cm^−2^, displaying the Faradaic efficiency as a function of time for the U‐NC and f) the S‐NC. d) The electrolyzer polarization curves of U‐NC and S‐NC; g) (C_2+_/C_1_) product FE‐ratio for both catalysts.

The loaded GDEs (loading of about 1 mg${}_{\mathrm{Cu}{}_{2}O}$ cm^−2^ geometric area) were tested in a commercial 4‐chamber electrolyzer flow cell. The ambient CO_2_ pressure minimized mass transport limitations and enabled catalytic tests at high currents, as depicted in Figure 6 a. During testing, a constant current was applied for two hours for each current step in the range of 50 to 700 mA cm^−2^ in 1 m KHCO_3_. Figure 6 b,c shows the change in the FEs of the major CO_2_RR products with variation of the applied current density. Both catalysts exhibited a high selectivity towards CO_2_RR, exceeding 70 % FE at 200 mA cm^−2^. Indeed, the U‐NC displayed an exceptionally high efficiency for C_2+_ products, in respect to our previous H‐Cell results, which increased with the applied current to a maximum of 59 % at 300 mA cm^−2^.

Again, our data evidenced a pronounced support effect. The S‐NCs produced mainly HCOO^−^ and CO up to a current density of 400 mA cm^−2^. Currents above 400 mA cm^−2^ favored C_2+_ selectivity, yet also caused the HER to dominate the overall faradaic processes, which resulted in a decrease in CO_2_RR selectivity. The electrolyzer results are in excellent agreement with our H‐Cell results, confirming the significant shift in the experimental CO_2_RR selectivity caused by the carbon support (Figure 6 g).

Since the electrolyzer polarization curves showed almost identical catalytic reactivity for both catalysts, we exclude overpotential as the origin of the experimental selectivity differences (see Figure 6 d).

Next, during stability tests over 40 hours at 300 mA cm^−2^, we uncovered a remarkably constant product selectivity for the U‐NC, whereas the S‐NC displayed a clear FE variation with time: the initially high FEs of CO and HCOO^−^ were continuously dropping, whereas the C_2+_ selectivity was constantly rising. Again, the two measured cathode potentials of the U‐NC and S‐NC during the 40 h stability tests showed no obvious differences that could account for the observed behavior (Figure S13). We carried out SEM imaging of the GDEs after the reaction to investigate the respective surface evolution of the two catalysts (Figure S11). While the U‐NC formed a rough, continuous surface after testing, the S‐NC displayed the presence of highly dispersed spherical Cu particles after the polarization test, which transformed into irregular Cu aggregates after the 40 h stability test. Note that the newly formed spherical Cu NPs were smaller than the initial Cu_2_O nanocubes. We suspect that the high mobility of these Cu nanoparticles on the carbon support surface is aiding in the observed morphological evolution. The agglomeration and growth of the reaction‐generated small Cu NPs results in the formation of larger particles decreasing their effective dispersion. It is conceivable that such a lowered dispersion contributed to our experimental data, accounting for the gradual change in the faradaic efficiency values, eventually matching those of the unsupported Cu nanoparticles. Moreover, recent studies have discussed similar morphological changes for systems of unsupported, metallic Cu cubes^24^ and supported oxidized copper particles.^25^ Interestingly, the first study shows a temporal decrease in C_2+_ efficiency, caused by a potential‐driven structural degradation of metallic Cu cubes and loss of the (100) facet during CO_2_RR, whereas the second study reports on a temporal increase of C_2+_ efficiency, caused by fragmentation and successive reconstruction resulting in a boundary‐rich Cu structure. This shows the need for careful distinction between effects of exposed crystal facets and the abundance of defects for shaped CuO_*x*_ catalysts in CO_2_RR, which we help to address here.

To trace this particle growth in more detail, we performed stepwise SEM analyses after of 4 and 20 hours of constant electrolysis at 300 mA cm^−2^ using the S‐NCs (Figure S12). While already after 4 hours the presence of emerging Cu particle aggregates was visible, we could also observe their precursors, that is, very small, isolated particles (indicated by a red cycle in Figure S12). We note that these tiny Cu seed particles were considerably smaller than the initial Cu_2_O nanocubes, suggesting a partial break‐up of the original Cu_2_O cubes during the catalytic reaction and electrochemical Cu_2_O reduction process.

## Conclusion

This contribution explored and aimed to identify chemical and structural factors that control the experimentally observed catalytic selectivity of the CO_2_RR. More precisely, this study traced the individual faradaic product efficiencies over time and linked their evolution to changes in the chemical state at the surface and bulk and in the catalyst morphology (see Figure S14). To achieve this goal, Cu_2_O cubes of nanometer‐sized dimensions were chosen as the catalytic active phase. Our main conclusions are as follows:

- Dispersion of the Cu_2_O cubes on a high surface area carbon support drastically shifted their faradaic efficiencies from C_2+_ towards C_1_ products. Hence, the otherwise identical Cu_2_O cubes exhibited a pronounced support effect, which we attribute, at least in part, to a larger interparticle distance that makes readsorption of CO and dimerization less likely.
- Tracking experiments using X‐ray diffraction and spectroscopy suggested that the initial Cu_2_O single phase gradually disappeared and gave way to metallic Cu nanoparticles. This process proceeded, however, slower than anticipated, and a noticeable fraction (up to 20 %) of Cu^I^ was present in the sample even after several hours of continuous CO_2_ electroreduction according to our *operando* XAS data. The surface of the initial oxidic nanocubes, however, reduced on shorter time scales (XPS). It also led us to a conclusion that the emergence of defects in the Cu lattice during the reduction of the nanocubes largely contributes to the observed stable efficiency patterns.
- The supported Cu_2_O cubes exhibit a significant evolution in their faradaic efficiencies toward those of the unsupported Cu_2_O cubes over a 40 h electrolyzer test. Concomitant tracking of the catalyst state led us to conclude that carbon‐dispersed Cu_2_O cubes are morphologically unstable, generate small seed particles, which subsequently agglomerate and grow into a structure that resembles the unsupported sample. As a result of this evolution, the efficiencies of C_2+_ products increase, while those of C_1_ products decline.

In all, our present data and conclusions demonstrate the impact that the nature of a support (metallic self‐support or carbon support) can have on the morphological stability and resulting catalytic product yields.

## Conflict of interest

The authors declare no conflict of interest.

## Supporting information

As a service to our authors and readers, this journal provides supporting information supplied by the authors. Such materials are peer reviewed and may be re‐organized for online delivery, but are not copy‐edited or typeset. Technical support issues arising from supporting information (other than missing files) should be addressed to the authors.

Supplementary
