# Supplementary material for: Electrocatalytic CO2 Reduction on CuOx Nanocubes: Tracking the Evolution of Chemical State, Geometric Structure, and Catalytic Selectivity using Operando Spectroscopy
Source: Angew Chem Int Ed Engl. 2020 Aug 13;59(41):17974–83. doi: 10.1002/anie.202007136 (PMC7590092; doi:10.1002/anie.202007136)
Supplement: Supplementary file 1 — Supplementary [file ANIE-59-17974-s001.pdf]

Angewandte Chemie
Eine Zeitschrift der Gesellschaft Deutscher Chemiker

Supporting Information

Electrocatalytic CO_2 Reduction on CuO_x Nanocubes: Tracking the Evolution of Chemical State, Geometric Structure, and Catalytic Selectivity using Operando Spectroscopy

Tim Möller, Fabian Scholten, Trung Ngo Thanh, Ilya Sinev, Janis Timoshenko, Xingli Wang, Zarko Jovanov, Manuel Gliech, Beatriz Roldan Cuenya, Ana Sofia Varela,* and Peter Strasser**

anie_202007136_sm_miscellaneous_information.pdf

1. Equations for product quantification

Equation S1. Production Rate of Gas Products

Equation S2. Faradaic Efficiency of Gas Products

Equation S3. Production Rate of Liquid Products

Equation S4. Faradaic Efficiency of Liquid Products

Equation S5. Partial current density

Equation S6. RHE potential

2. Experimental Details

2.1 Catalyst synthesis

2.2 Material Characterization

2.3 Electrochemical Characterization

2.4 Product analysis

3. Physical and Chemical Characterization

Figure S1: S-NC (44 wt%) catalyst characterization in H-Cell.

Table S1: Catalyst loading during CO_2RR in H-Cell

Figure S2. Morphological investigation of as-prepared and after-reaction U-NC and S-NC catalysts.

Figure S3: Lead Under Potential Deposition (Pb-UPD)

Figure S4: Activity of the substrate during CO_2RR in H-Cell

Figure S5: Geometric and mass activity during CO_2RR for all tested catalysts in H-Cell

Figure S6: Stability tests of CO_2RR in H-Cell for U-NC and S-NC (23 wt%) catalysts

Figure S7: Post-reaction XRD measurements of the supported (23 wt%) and unsupported catalyst

Figure S8: Cu-AES measurements of the as-prepared supported (23 wt%) and unsupported catalyst.

Figure S9: XANES and EXAFS spectra of the Cu_2O cubes and references

Figure S10: Fitting of EXAFS spectra

Table S2: Evolution of coordination numbers and interatomic distances from EXAFS

Figure S11: SEM measurements after Flow-Cell testing

Figure S12: SEM measurements after Flow-Cell testing at 300 mA cm^{-2} on the supported particles

Figure S13: Temporal evolution of potential during stability testing in the Flow-Cell

Figure S14: Schematic representation of the morphological and structural changes of the shaped cubic Cu2O during CO_2RR in a flow-electrolyzer.

1. Equations for product quantification

Equation S1. Production Rate of Gas Products

$$\dot{n}_x = \frac{\dot{V} * C_x}{A * V_M}$$

$\dot{n}_x$: Generation rate of the product x / mol s^{-1} cm^{-2}

$\dot{V}$: CO_2 gas flow rate / L s^{-1}

C: Volumefraction of the product x detected by GC

A: Geometric area of the electrode / cm^{-2}

V_M: molar Volume / 22.4 L mol^{-1}

Equation S2. Faradaic Efficiency of Gas Products

$$FE_x = \frac{\dot{n}_x * z_x * F}{j_{total}} * 100\%$$

FE_x: Faradaic Efficiency of the product x / %

$\dot{n}_x$: Generation rate of the product x / mol s^{-1} cm^{-2}

z_x: electrons transferred for reduction to product x

F: Faradaic Constant / C mol^{-1}

j_{total}: Total current density during CO_2 bulk electrolysis / A cm^{-2}

Equation S3. Production Rate of Liquid Products

$$\dot{n}_x = \frac{V * \Delta C_x}{A * \Delta t}$$

$\dot{n}_x$: Generation rate of the product x / mol s^{-1} cm^{-2}

V: Volume of the electrolyte / L

ΔC_x: Accumulated concentration of the product x detected by HPLC or liquid GC / mol L^{-1}

A: Geometric area of the electrode / cm^2

Δt: Reaction time at const. current or potential / s

Equation S4. Faradaic Efficiency of Liquid Products

$$FE_x = \frac{V * \Delta C_x * z_x * F}{\Delta Q} * 100\%$$

FE_x: Faradaic Efficiency of the product x / %

V: Volume of the electrolyte / L

ΔC_x: Accumulated concentration of the product x detected by HPLC or liquid GC / mol L^{-1}

z_x: electrons transferred for reduction to product x

ΔQ: Total charge transfer during the electrolysis at const. potential or current / C

F: Faradaic Constant / C mol^{-1}

Equation S5. Partial current density

$$j_x = \frac{FE_x * j_{total}}{100}$$

j_x: Partial current density / Acm^{-2}*

FE_x: Faradaic Efficiency of the product x / %

j_{total}: total current density / Acm^{-2}*

Equation S6. RHE potential

$$E_{RHE} = E_{Ref} + E_{Ag/AgCl} + 0.059 * pH + U * I$$

E_{RHE}: RHE potential / V

E_{Ref}: Applied potential against the reference electrode / V

$E_{Ag/AgCl}$: Potential of the reference electrode measured against NHE (0.21 V) / V

pH: pH-value of the electrolyte

U: Ohmic resistance between working and reference electrode / Ω

I: Total Current of the experiment / A

2. Experimental Details

2.1 Catalyst synthesis.

Ambient pressure reduction and precipitation of Cu salts in the presence of ascorbic acid was used to prepare unsupported cubic Cu_2O nanoparticles (NPs). Subsequently, deposition of weighted amounts of dispersed NPs on a high surface area Vulcan® carbon resulted in supported Cu_2O nanocubes with varying catalyst weight loadings.

Cu_2O nanoparticles (NPs) were synthesized according to a prior work.[1] In brief, a dilute solution of $CuCl_2$ was prepared by adding 7 mL of a 0.1 M $CuCl_2$*$2H_2O$ (Sigma-Aldrich, ACS reagent) solution to 280 mL 18 MΩ Milli-Q-water®. After a short stirring time of about 5 minutes, a solution of sodium hydroxide (21 mL, 0.2 M, Sigma-Aldrich, semiconductor grade) was poured into the reaction mixture followed by addition of a solution of l-ascorbic acid (14 mL, 0.1 M, Sigma-Aldrich, ACS reagent). The reaction mixture was stirred during the following hour. After this time, the Cu_2O NPs were precipitated by centrifugation. The raw product was cleaned with water and ethanol.

To support the NPs on a conductive carbon support, Vulcan® XC-27R (Cabot) was dispersed in cyclohexane using a sonication horn (Branson Corp.). After 30 minutes, Cu_2O NPs were added to the suspension and the sonication was continued for one hour. Afterwards, the dispersion was stirred overnight to allow the particles to settle on the support. The catalyst was separated from the liquid phase by centrifugation, followed by washing it three times with ethanol.

2.2 Material Characterization

XRD-patterns of the supported and unsupported catalysts were collected using a D8 Advance Diffractometer (Bruker) equipped with a Lynx Eye Detector and KFL Cu 2K X ray tube. Powder samples were analyzed in a 2θ range of 25-80 ° with a step size of 0.07° and a collection time of seven seconds per step. Film samples were analyzed by grazing incidence XRD (GI-XRD) in an equal device but in a 2θ range of 20-80° with a step size of 0.06°, a collection time of 40 s per step and an incident angle of 1°.

The loading of the catalyst on the carbon support was calculated from the metal content in 10 mg of catalyst dissolved in a in a mixture of nitric, hydrochloric and sulfuric acid (1:3:1). The Cu concentration in the resulting solution was detected by Inductively Coupled Plasma Optical

Emission Spectrometry (ICP-OES) using an atomic emission spectrometer (715-ES, Varian). Comparison to Cu standards of known concentration allowed the determination of the respective Cu concentration.

Transmission Electron Microscopy (TEM) measurements were performed using a Tecnai G2 microscope 20 S-Twin with a LaB6-cathode at 200 kV accelerating voltage (ZELMI Centrum, Technical University Berlin). The supported and unsupported samples were dispersed in ethanol and drop-casted onto Cu-grids. For evaluation of the size-distribution, roughly 100 particles were counted, using a software (ImageJ2x) to process the images.

Scanning Electron Microscopy (SEM) measurements were performed by using a JEOL 7401F instrument equipped with both SEI and a COMPO detector at an acceleration voltage of 10 kV. For imaging of as prepared samples either on glassy carbon plates or gas diffusion layers, routine electrode preparation, as done for electrochemical testing was undertaken. For analysis of electrodes after CO_2RR, the samples were rinsed with milli-Q water after electrochemistry to remove remaining $KHCO_3$ and dried in a nitrogen stream prior to conducting the microscopy analysis.

X-ray absorption spectroscopy (XAS) measurements were carried out at the CLÆSS beamline of Alba synchrotron radiation facility (Barcelona, Spain). A Pt-coated toroid mirror was used to reject higher harmonics in the X-ray beam and to focus it to a 500 x 500 μm^2 spot on the sample. The *operando* X-ray absorption data were recorded at the CuK- edge (8979 eV) at room temperature in fluorescence mode using a single-channel silicon drift detector (SDD). A home-built *operando* electrochemical cell was used, with a Pt foil counter electrode and Ag/AgCl reference electrode. The Cu_2O NP powder was deposited on a graphite paper disc (Sigracet 24 BC, SGL CARBON GmbH) by filtration from a slurry containing Nafion (0.1 v/v %) as a binding agent and dissolved in ethanol. Due to low porosity of the paper, the catalyst stays on one side, while the other side stays unmodified. The paper disc was mounted in the *operando* cell so that the unmodified side was facing out, while the side containing the catalyst layer was in contact with the electrolyte. Using this approach, we successfully avoided intensity losses in the incident X-ray beam as well as fluorescence radiation emitted from the sample while passing through the electrolyte layer. The sample was measured in as prepared state, at -0.66 and at -0.95 V vs. RHE. The as prepared sample

as well as commercial CuO, Cu_2O and Cu foil references were measured in transmission mode. At each applied potential, the measurements were carried out until no further changes in the XAS spectra were observed. Details of the XAS cell can be found in our recent publication.[2]

All quasi in situ X-Ray photon spectroscopy (XPS) measurements were carried out with a commercial Phoibus 100 (SPECS GmbH) analyzer using an Al anode of the XR 50 X-ray source (SPECS GmbH). To prevent samples from re-oxidation in air after running the CO_2RR, our customized electrochemical cell was directly attached to the UHV XPS-analysis system, and sample transfer took place under a protective pressurized (p=1.05 bar) Ar atmosphere. All samples were rinsed with 10 ml of N_2-bubbled (1 hour) deionized water (R=18.2 MΩ) after electrochemistry to clean the samples from the residual $KHCO_3$ electrolyte. All reactions were run for 1h before acquiring the respective XPS spectra. The XPS data were aligned to the binding energy of carbon (E_b= 284.9 eV) for all samples, resulting in a binding energy of the Cu 2p 3/2 spectra of E_b=932.6 eV which is in agreement with the literature.[3]

2.3 Electrochemical characterization

The first set of electrochemical measurements was carried out in a custom-made two-compartment H-type cell, in which the working and counter electrodes were separated by a 212 Nafion® membrane. For long-term testing an anion exchange membrane (Selemion AMV, AGC Engineering Co., LTD.) used. The glassware was cleaned in a NOCHROMIX® bath containing sulfuric acid and afterwards in concentrated HNO_3 for 1 h, respectively. To remove remaining acid, the cell was rinsed extensively and sonicated submerged in ultra-pure water several times.

Each compartment of the cell was filled with 40 mL of 0.1 M $KHCO_3$ (40 mL, Sigma-Aldrich, ≥99.95%). Before and during the electrochemical reaction the electrolyte was purged continuously with CO_2 (30 mL min^{-1}, 4.5N) from the bottom of the cell, which was regulated using a mass flow controller (Bronkhorst).

A platinum mesh 100 (Sigma-Aldrich 99.9%) was used as counter electrode (CE) and a leak-free Ag/AgCl electrode as reference electrode (Multi Channel Systems MCS GmbH). Control repeat experiments using an IrO_x counter electrode yielded identical results, suggesting that dissolved Pt ions at the CE did not affect the measurement at the working electrode (WE). The WE was prepared by drop casting 50 μL of the catalysts ink onto 1 cm^2 of a glassy carbon plate. The ink was prepared by dispersing either 15 mg of the supported or 4 mg of the unsupported nanoparticles

in a mixture of 1.6 mL of ultrapure water, 300 µL of iso-propanol and 100 µL Nafion® solution (Sigma-Aldrich, 5%). The mass of the nanocubes deposited on the glassy carbon plate working electrodes is given in Table S1. For each measurement, a new electrode was prepared to avoid influences of adsorbates or catalyst changes on the following experiments. Before ink deposition, the glassy carbon plates were mechanically polished with alumina paste, followed by sonication in ultra-pure water and acetone.

Every measurement started with a linear voltammetric sweep, performed with a scan rate of −5 mV/s between E = +0.05 V_{RHE} and the working potential (between -0.6 V_{RHE} and -1.0V_{RHE}) and followed by a chronoamperometric step for one hour. All reported potentials are corrected for Ohmic drop, which was determined by electrochemical impedance spectroscopy (EIS). EC-Lab software was used to automatically correct 50% of the Ohmic drop, the remaining 50% was corrected manually.

To evaluate the difference in accessible surface area of the electrochemically active Cu-sites, for the unsupported and supported catalysts (23 wt% metal loading), lead under potential deposition (Pb-UPD) was performed on samples which were reduced for five hours at -0.86 V vs. RHE in CO_2 saturated 0.1 M $KHCO_3$ prior to deposition. The deposition was performed in a degassed (N_2) solution of 0.01 M $Pb(ClO_4)_2$*xH_2O (Aldrich, ≥ 99.995%) in 0.1 M $HClO_4$ (Aldrich, 70%) . The cathode-potential was cycled in between -0.40 V to -0.05 V vs Ag/AgCl at a scan rate of 10 mV s^{-1} until a stable cyclic voltammogram (CV) could be obtained. The charge of the anodic peak at -0.24 V vs Ag/AgCl, which represents the stripping of the Pb-monolayer from the Cu surface, was evaluated to compare both catalysts. In both cases, 100 µg of total particle mass was deposited on one cm^2 of the glassy carbon electrode.

In the second set of experiments, measurements at high current densities were performed in a commercial Micro-Flow-Cell (MFC) supplied by ElectroCell. The catalyst was spray-coated on the microporous layer (MPL) of a Freudenberg C2 gas diffusion layer (GDL). A geometric area of 3 cm^2 was coated to achieve a final metal loading of 1 about mg cm^{-2}, additionally 30 wt% of Nafion (relative to the total loading) was used as binder and for ionic conductivity of the catalyst-layer. 1 M $KHCO_3$ (500 mL, Sigma-Aldrich, BioUltra, ≥99.5%) was used as anolyte and catholyte, which were separated by an anion exchange membrane (Selemion AMV, AGC Engineering Co., LTD.). As anode a commercial IrO_x-coated Ti sheet with an active area of 10 cm^2 was used (ElectroCell).

Both electrolytes were cycled through each respective compartment at 100 mL min^{-1} by using a peristaltic pump (PMP Ecoline, Cole-Parmer). The CO_2 (4.5N) was supplied at a rate of 50 mL min^{-1} to the cathode and was flown from the back of the GDL through the catalyst-layer. Measurements were performed galvanostatically for 2 h at each respective current during the catalytic tests, starting at 50 mA cm-^{2} and increasing stepwise towards higher values. Each galvanostatic step was followed by a PEIS measurement at OCV to account for the ohmic drop in calculations of IR-free RHE potentials.

2.4 Product analysis

After the gas stream passed the CO_2RR cell, it was directly introduced into a Gas Chromatograph (GC, Shimadzu GC 2014) equipped with a HayeSep Q and a HayeSep R column for product separation. In case of flow cell experiments, only a HayeSep D column was used for separation. A Flame Ionization Detector (FID, for CO, CO_2, CH_4 and C_2H_4) and a Thermal Conductivity Detector (TCD, for H_2) were used to quantify gaseous products.

Additionally, 2 mL of the catholyte were sampled after reaction at constant potential or current by an automatic aliquot sampling device. This sample was analyzed by the high performance liquid chromatograph (HPLC, Agilent 1200 series, Organic-Acid Resin column) to quantify carboxylic acids using a Refractive Index Detector (RID) and by the liquid injection gas chromatography (Shimadzu GC 2010 plus, SH-Stabilwax Capillary Column, FID Detector) to determine alcohol concentration.

3. Physical and chemical characterization of the catalyst

TEM and SAED characterization of the prepared materials

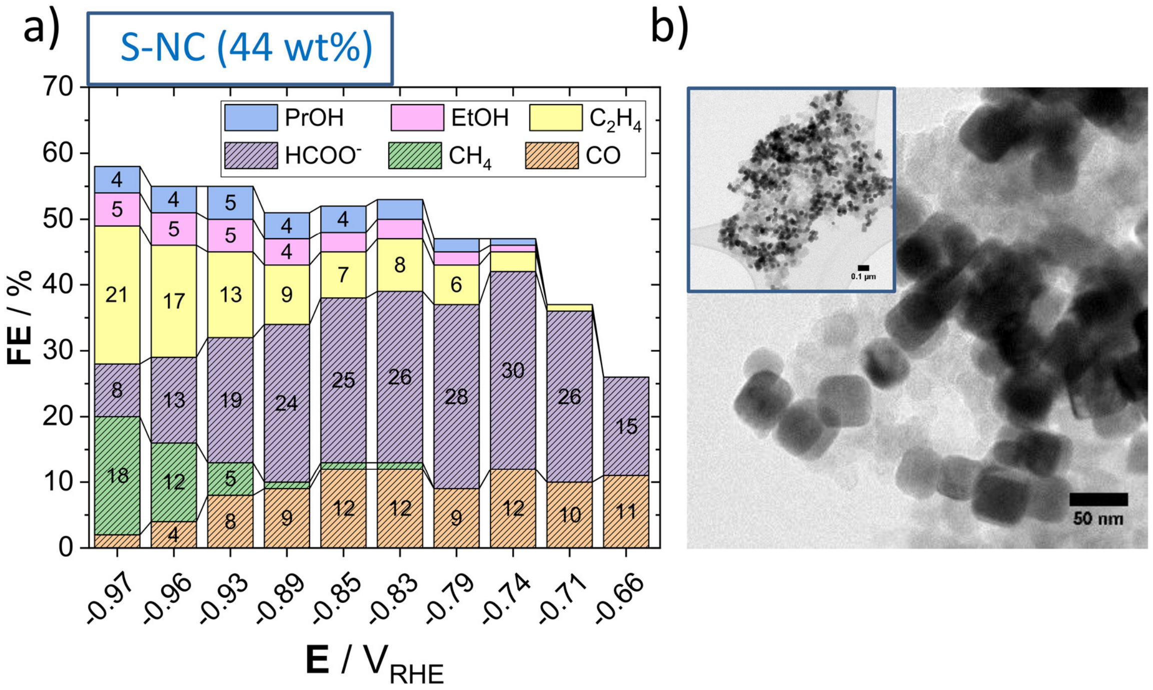

Figure S1. S-NC (44 wt%) catalyst characterization in H-Cell. (a) *Faradaic product efficiencies (FEs) as a function of IR-free applied electrode potential after one hour of reaction time. (b) Transmission electron microscopy (TEM) images of the S-NC (44 wt%) catalyst, a low magnification TEM is given in the inlet.*

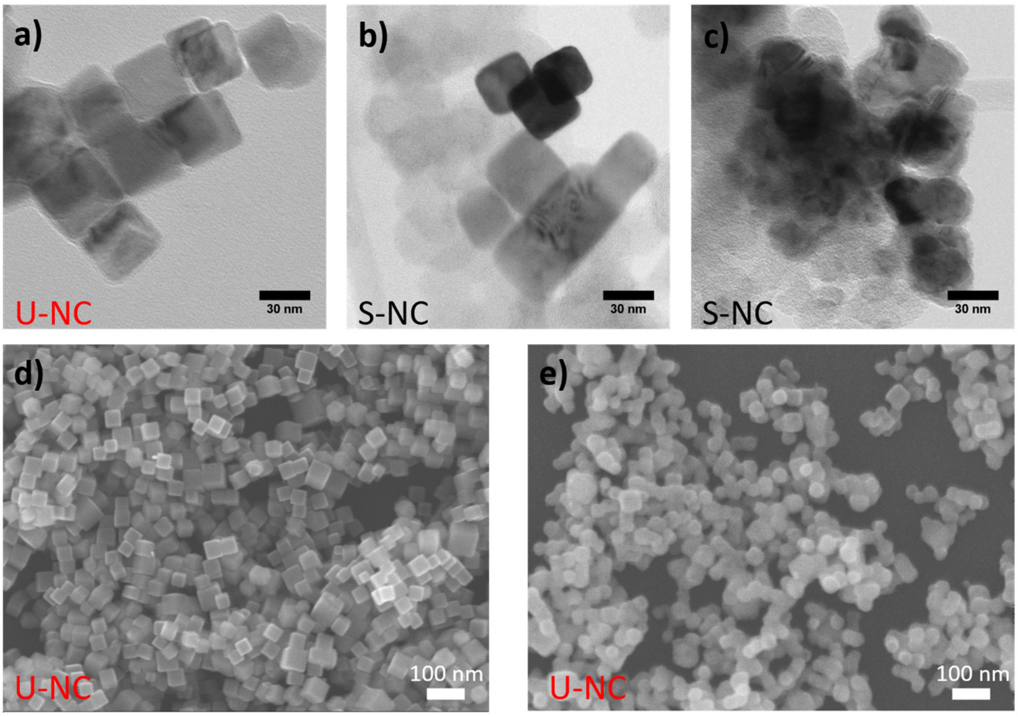

Figure S2. Morphological investigation of as-prepared and after-reaction U-NC and S-NC catalyts. *Transmission electron microscopy (TEM) images of the U-NC (a) and S-NC (b) catalyst in as prepared state and for the S-NC after CO2RR at an electode potential of -0.95 V_{RHE} in H-Cell (c). Scanning electron microscopy (SEM) images of the U-NC catalyst on a glassy carbon electrode, in as prepared state (d) and after CO2RR at an electode potential of -0.95 V_{RHE} in H-Cell (e). In case of the U-NC catalyst no Nafion was used and the loading was reduced to 20 µg cm^{-2} to allow a clearer imaging of the particles.Time of electrolysis was 60 min in all cases.*

Geometric mass loading of catalyst used in H-Cell experiments

	U-NC	23 wt% S-NC	44 wt% S-NC
Total amount of catalyst deposited on GC plate [μg/cm^2]	100	380	380
Total Cu_2O particle loading on GC plate [μg/cm^2]	100	87	167

Table S1. Catalyst loading during CO_2RR in H-Cell. Total amount of catalyst-powder and respective Cu_2O particles deposited on the glassy carbon (GC) plates for CO_2RR experiments in H-Cell.

Electrochemical characterization in H-Cell

Lead Under Potential Deposition (Pb-UPD)

In order to be able to compare catalytic activities on a real surface area basis, and include differences in roughness of the two catalysts in the discussion of their performances, we evaluated the real electrochemically accessible surface area of both supported, S-NCs, and unsupported Cu nanocubes, U-NCs, using a very specific and selective electrochemical Pb under potential deposition (Pb-UPD) technique. Under electrochemical UPD conditions, that is, at electrode potentials slightly more positive than their standard potential, Pb adatoms form a monolayer on metallic Cu surfaces in a 2-electron process. We assessed the real electrochemical surface area after five hours of continuing CO_2 electrolysis, when the surface of the Cu_2O nanocubes had chemically reduced to metallic Cu (*vide infra*). At identical geometric catalyst mass loadings, the anodic stripping charge of a single Pb monolayer deposited on the Cu nanocubes was considerably larger for the S-NCs (Figure S2) than for the U-NCs, suggesting a larger real active surface area. This is well in line with the notion of dispersed nanocubes on the surface of the carbon support with accessible surface atoms, as opposed to agglomerated Cu nanocubes in case of the unsupported catalyst. Assuming a hexagonal closed packed Pb monolayer, we can use the specific charge of 300 µC cm^{-2}, known from the literature,[4] to estimate the real surface area exhibited by the U-NC (0.8 cm^2) and S-NC (2.57 cm^2) catalyst.

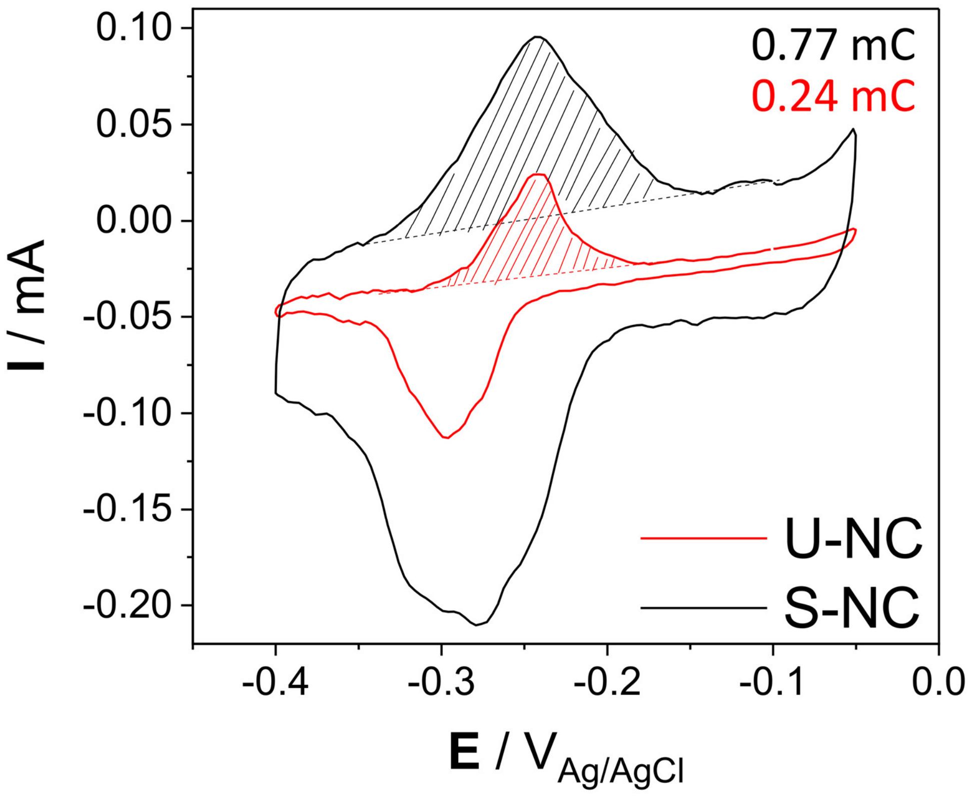

Figure S3. Lead Under Potential Deposition (Pb-UPD). Final CVs obtained for the unsupported nano cubes, U-NC, and supported nano cubes, S-NC, (23 wt%) catalyst after cycling in an aqueous solution of 0.01 M $Pb(ClO_4)_2$ and 0.1 M $HClO_4$ at 10 mV s^{-1} in between -0.40 V and -0.05 V vs Ag/AgCl. Prior to Pb-UPD both samples have been reduced for 5 h at -0.86 V_{RHE} during CO_2RR to ensure a purely metallic material. The given value represents the charge derived from the anodic stripping peak at -0.24 V vs Ag/AgCl, indicated by the shaded area.

Activity of the substrate during CO_2RR

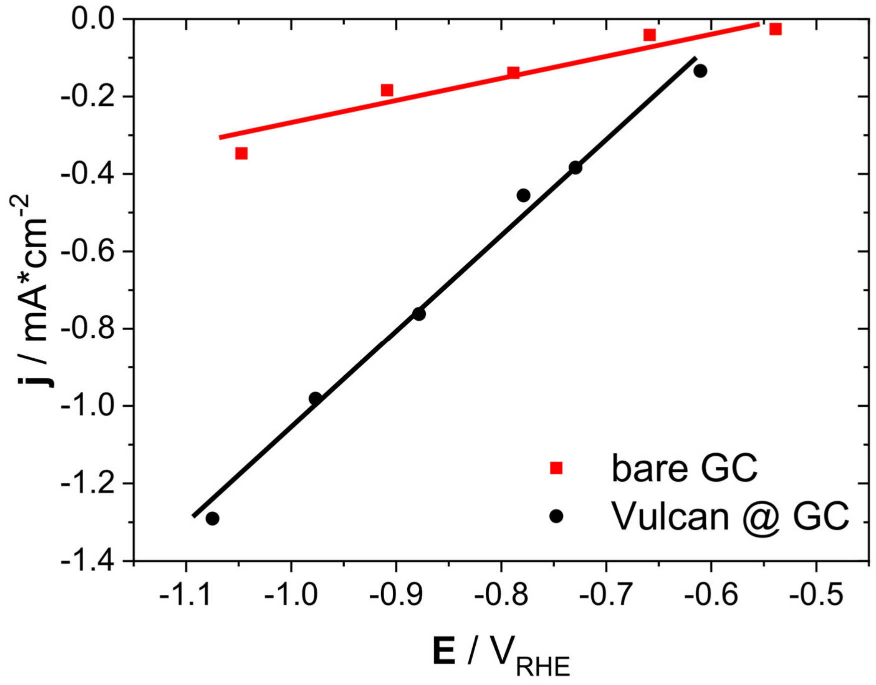

Figure S4. Activity of the substrate during CO_2RR in H-Cell. Current density achieved by using the bare glassy carbon plate (GC) or 300 µg cm^{-2} of Vulcan carbon deposited on a GC plate. The conditions were equal to the CO_2RR tests including the Cu_2O particles. Lines to guide the eye.

Activity during CO_2RR of all investigated catalysts in H-Cell

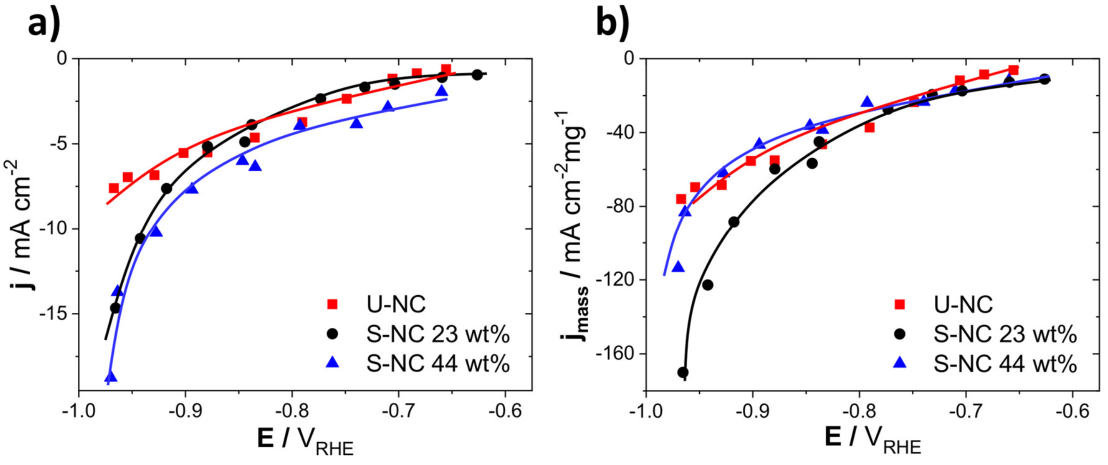

Figure S5. Geometric and mass activity during CO_2RR for all tested catalysts in H-Cell. Current density normalized to geometric area (a) and to deposited mass of Cu (b) after one hour of constant potential. The reported value resulted from the averaged current during the last minute of reaction. Lines to guide the eye.

Long-term stability tests for U-NC and S-NC catalysts.

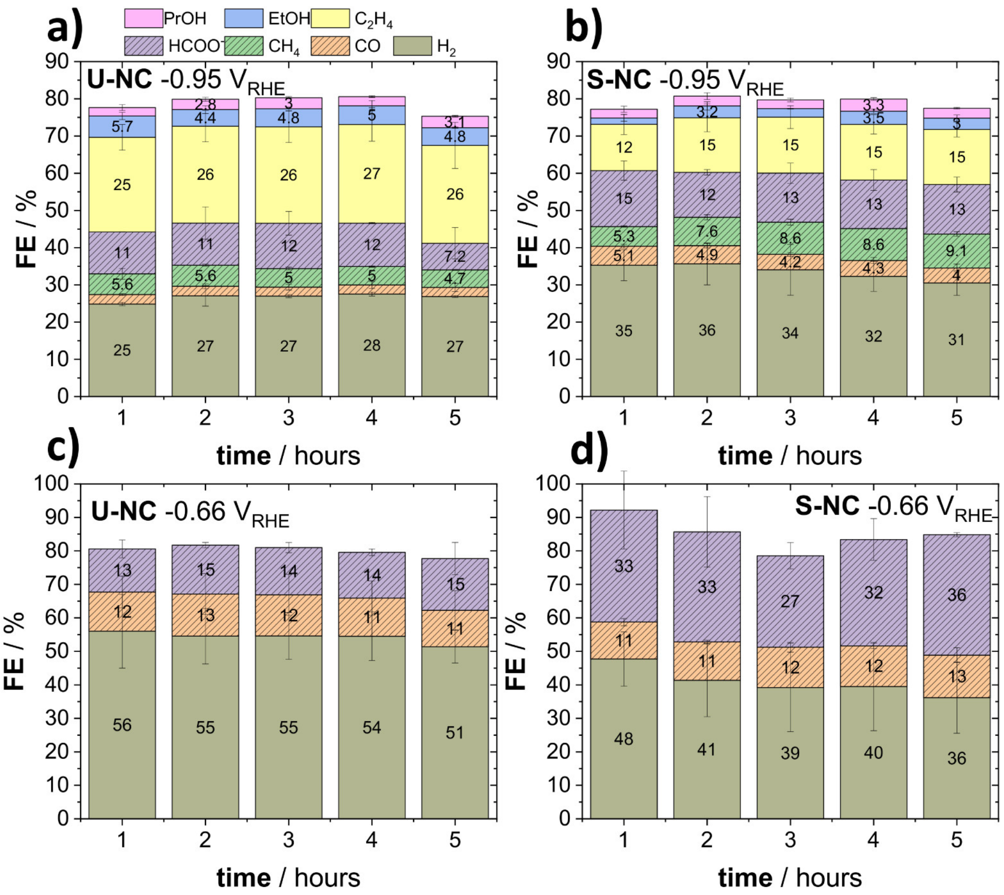

Figure S6. Stability tests of CO_2RR in H-Cell for U-NC and S-NC (23 wt%) catalysts. Faradaic efficiency during CO_2RR over 5 hours for the U-NC catalyst at -0.95 V_{RHE} (a) and -0.66 V_{RHE} (c), as well as for S-NC catalyst at -0.95 V_{RHE} (b) and -0.66 V_{RHE} (d). Values are the average of 2 independent measurements and error bars represent the respective standard deviation. The electrolyte is 0.1 M $KHCO_3$ saturated with CO_2 (pH = 6.8).

Post-reaction and *operando* characterization of the catalyst.

Post-reaction X-Ray Diffraction (XRD):

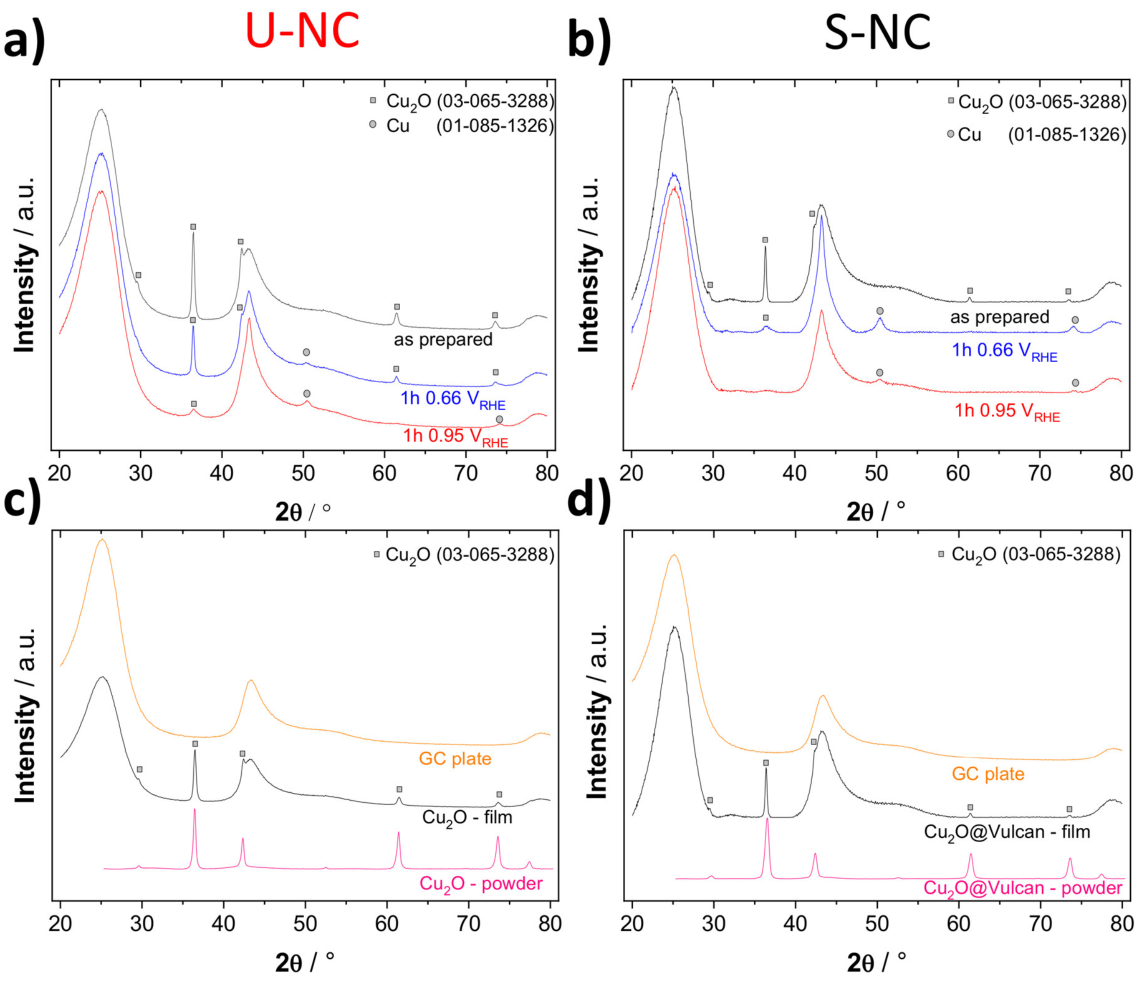

Figure S7. Post-reaction XRD measurements of the supported (23 wt%) and unsupported catalyst. XRD measurements of the unsupported (a) and 23 wt% supported catalyst (b) on GC plate as prepared (black), after one hour of CO2RR conditions at -0.66 V_{RHE} (blue) and at -0.95 V_{RHE} (red). Comparison of a XRD measurement for the GC plate substrate (orange) and catalyst powder (pink) for the unsupported catalyst (c) and the 23 wt% catalyst (d) illustrating the origin of the film-XRD pattern of the respective catalyst (black).

As-prepared Cu-AES:

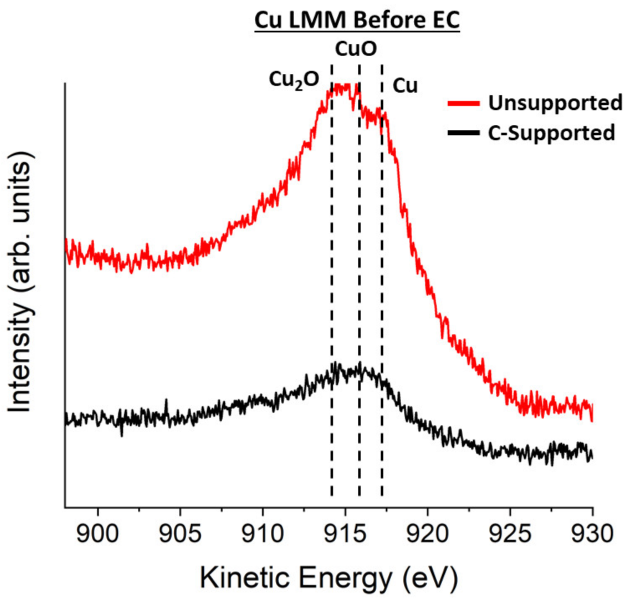

Figure S8. Cu-AES measurements of the as-prepared supported (23 wt%) and unsupported catalyst.

X-ray Absorption Spectroscopy (XAS – XANES, EXAFS)

The ex situ XAS fingerprints of the as-prepared U-NCs (see Figure S7) match those of the Cu_2O reference material almost perfectly, suggesting similarity in both the Cu chemical state and the local coordination. The XANES spectrum (Figure S7a) showed an intense pre-edge feature at 8981.1 eV typical for Cu_2O (1s-3d transition). Similarly, the first feature above the edge has its maximum at 8995.5 eV and is considerably less intense than the corresponding characteristic feature of the CuO reference. The Fourier transform of the EXAFS spectrum (Figure S7b) also displayed the characteristic peaks of Cu_2O, namely at 1.4 and 2.8 Å (uncorrected for phase shift). The former peaks along with the structure, well seen between ca. 3.5 and 5.5 Å, indicate a well-developed long-range order in the sample.

The slightly higher intensity of the pre-edge feature in the *operando* XANES data (Figure 5) as compared to that of a metallic Cu reference is a signature of self-absorption effect. However, by comparing the results of quantitative XANES and EXAFS analysis for the as-prepared sample (for which both fluorescence and transmission data were available, where the latter are not affected by self-absorption, see Table S2), the obtained values agree within the uncertainties of our analysis, confirming that self-absorption does not affect the conclusions.

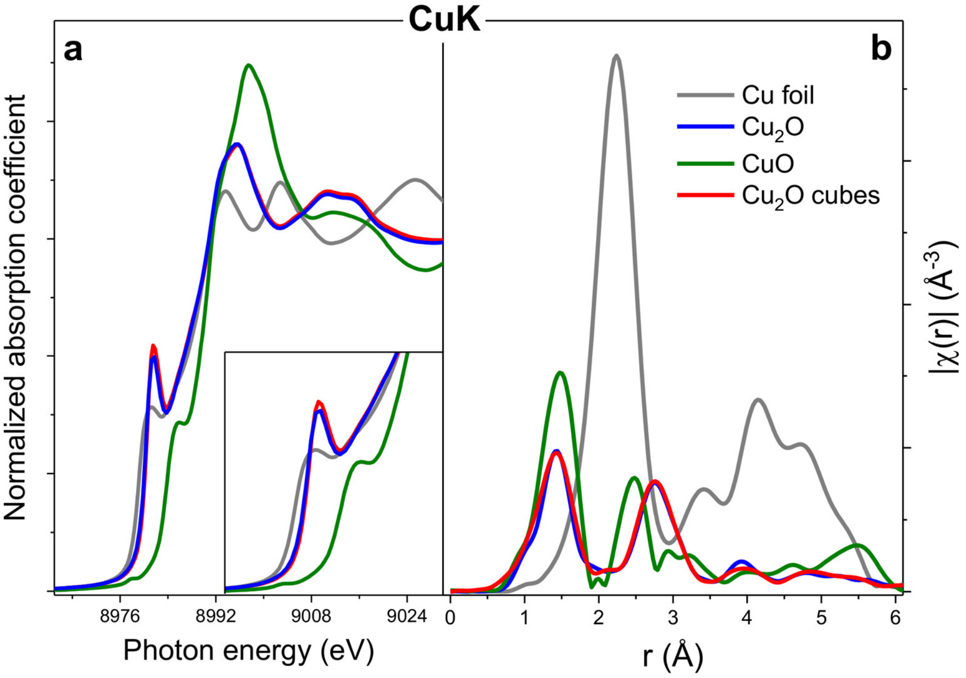

Figure S9. XANES and EXAFS spectra of the Cu_2O cubes and references. The initial as-prepared state of Cu_2O cube sample is displayed and compared with references. (a) Cu K-edge X-ray absorption near edge structure (XANES) spectra. The inset shows the pre-edge feature. (b) – Fourier transformed Cu K-edge extended X-ray absorption fine structure (EXAFS) spectra.

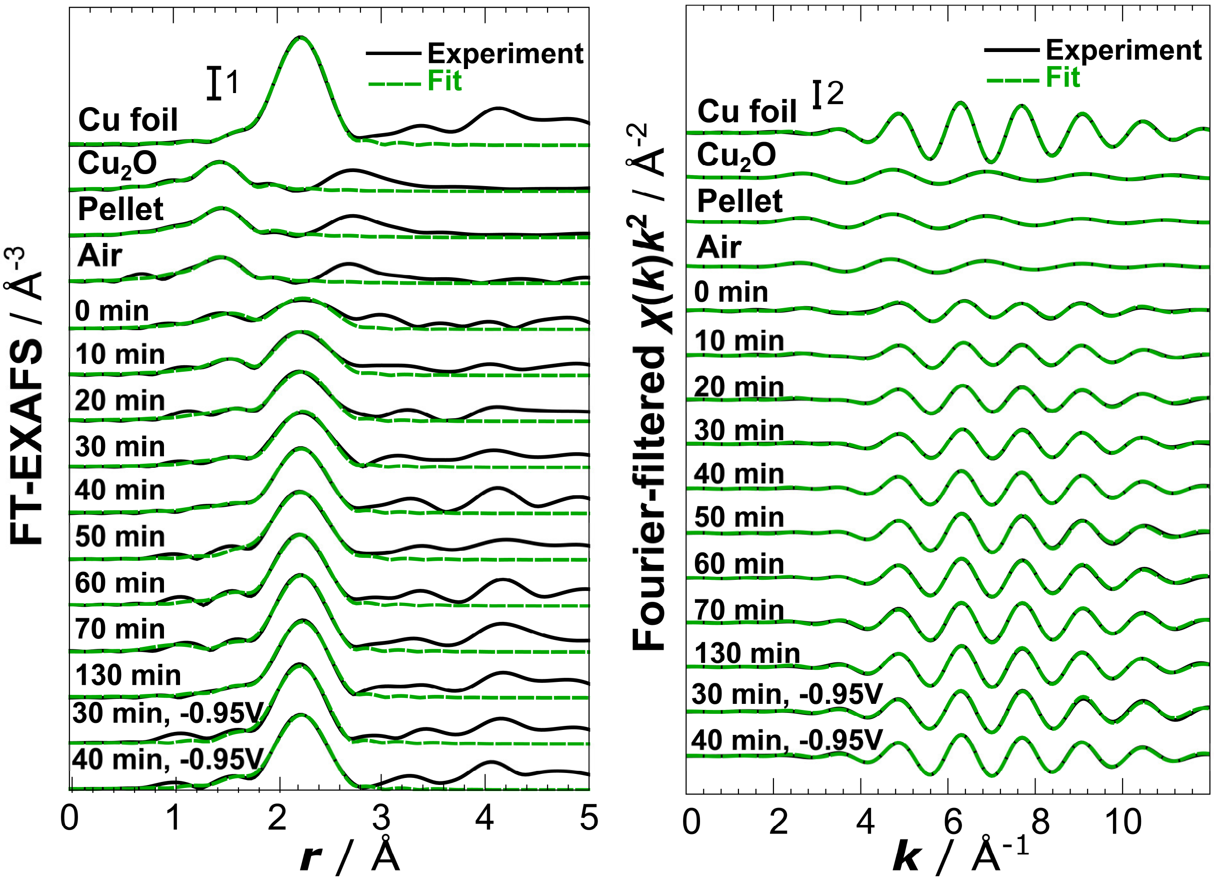

Figure S10. Fits of EXAFS spectra of the Cu_2O cubes and references in *r* and *k*-spaces Spectra for the initial as-prepared state of Cu_2O cube sample measured in transmission ("Pellet") and in fluorescence mode in electrochemical cell without electrolyte ("Air") are compared with the spectra for reference samples (Cu foil and Cu_2O) and with time-dependent *operando* EXAFS data collected at -0.66V_{RHE} (spectra for 0 min - 130 min) and at -.95 V_{RHE}. Fitting of EXAFS spectra $\chi(k)k^2$ was carried out in *r*-space in the range from 1.0 Å up to 2.8 Å (2.1 Å for Cu_2O). Fourier transform was carried out in the k range from 3 Å^{-1} up to 12 Å^{-1}.

Sample	N_{Cu-Cu}	R_{Cu-Cu} (Å)	σ^2_{Cu-Cu} (Å^2)	N_{Cu-O}	R_{Cu-O} (Å)	σ^2_{Cu-O} (Å^2)	ΔE_0 (eV)	R-factor
**Cu foil**	12	2.541(3)	0.009(4)	0	-	-	2.0(5)	0.002
**Cu_2O**	0	-	-	2	1.836(8)	0.002(1)	1.1(1)	0.011
**As-prepared (pellet, transmission)**	0	-	-	2.2(2)	1.848(6)	0.003(1)	2(1)	0.006
**As-prepared (air, fluorescence)**	0	-	-	2.1(2)	1.846(9)	0.003(1)	0(1)	0.011
**-0.66 V_{RHE}, 0 min**	3.6(8)	2.56(1)	0.009(2)	1.1(4)	1.90(3)	0.003(5)	7(2)	0.028
**-0.66 V_{RHE}, 10 min**	4.6(6)	2.553(8)	0.008(1)	0.8(3)	1.88(2)	0.001(3)	5(1)	0.01
**-0.66 V_{RHE}, 20 min**	5.3(8)	2.545(9)	0.000(1)	0.8(5)	1.89(3)	0.005(9)	4(1)	0.014
**-0.66 V_{RHE}, 30 min**	5.5(6)	2.548(7)	0.0078(9)	0.6(3)	1.87(3)	0.004(8)	4(1)	0.008
**-0.66 V_{RHE}, 40 min**	6.6(3)	2.542(3)	0.0082(4)	0.4(2)	1.86(2)	0.002(5)	3(1)	0.002
**-0.66 V_{RHE}, 50 min**	7.6(7)	2.54(6)	0.0090(8)	0.4(3)	1.89(4)	0.001(9)	2(1)	0.005
**-0.66 V_{RHE}, 60 min**	7.2(8)	2.536(7)	0.0082(9)	0.4(4)	1.90(4)	0.01(1)	3(1)	0.009
**-0.66 V_{RHE}, 70 min**	7.3(5)	2.539(5)	0.0077(6)	0.5(6)	1.86(6)	0.01(2)	2.6(8)	0.003
**-0.66 V_{RHE}, 130 min**	7.9(6)	2.545(5)	0.0084(7)	0.3(3)	1.91(5)	0.01(1)	3.3(8)	0.004
**-0.95 V_{RHE}, 30 min**	7(1)	2.53(1)	0.007(1)	0.2(6)	2.1 (1)	0.01(3)	1(2)	0.015
**-0.95 V_{RHE}, 40 min**	8.2(7)	2.543(6)	0.0088(8)	0.3(3)	1.92(5)	0.01(1)	3(1)	0.005

Table S2. Evolution of coordination numbers and interatomic distances from EXAFS. Coordination numbers (N), interatomic distances (R) and disorder factors (σ^2) of Cu-O and Cu-Cu nearest neighbors from EXAFS, as well as corrections to photoelectron reference energies ΔE_0.

Flow-Cell characterization

SEM images before and after reaction in Flow-cell

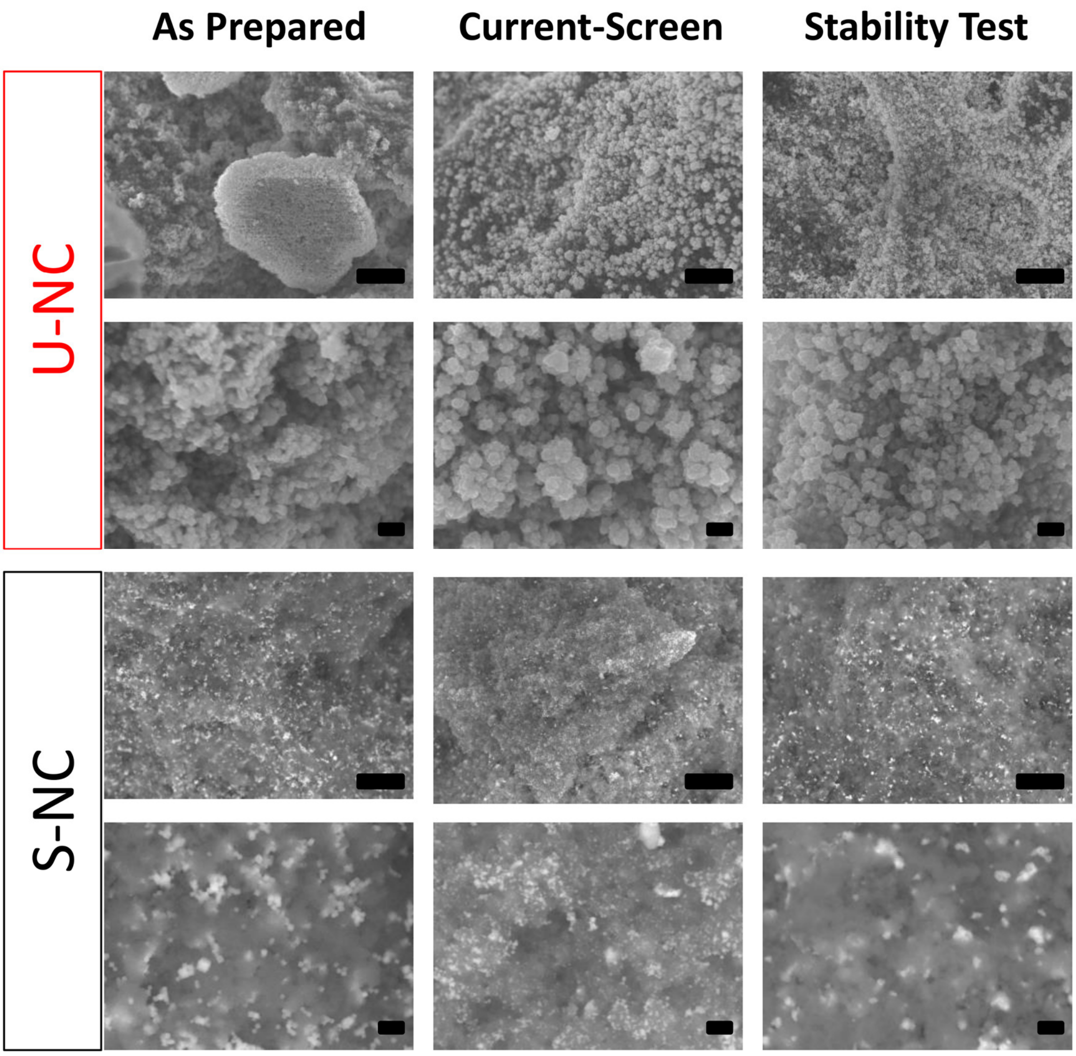

Figure S11. SEM measurements after Flow-Cell testing. SEM images of the unsupported particles (top two rows) and SEM-COMPO images of the supported particles (bottom two rows) in as prepared state, after the current-screen in between -50 to -700 mA cm^{-2} and 40 h stability test in the flow-cell at -300 mA cm^{-2}. Scale bars in the low-magnification images (first and third row) represent 2000 nm and represent 200 nm in the high-magnification images (second and fourth row).

SEM-COMPO images of the supported (23 wt% metal loading) catalyst after stability tests in the Flow-Cell.

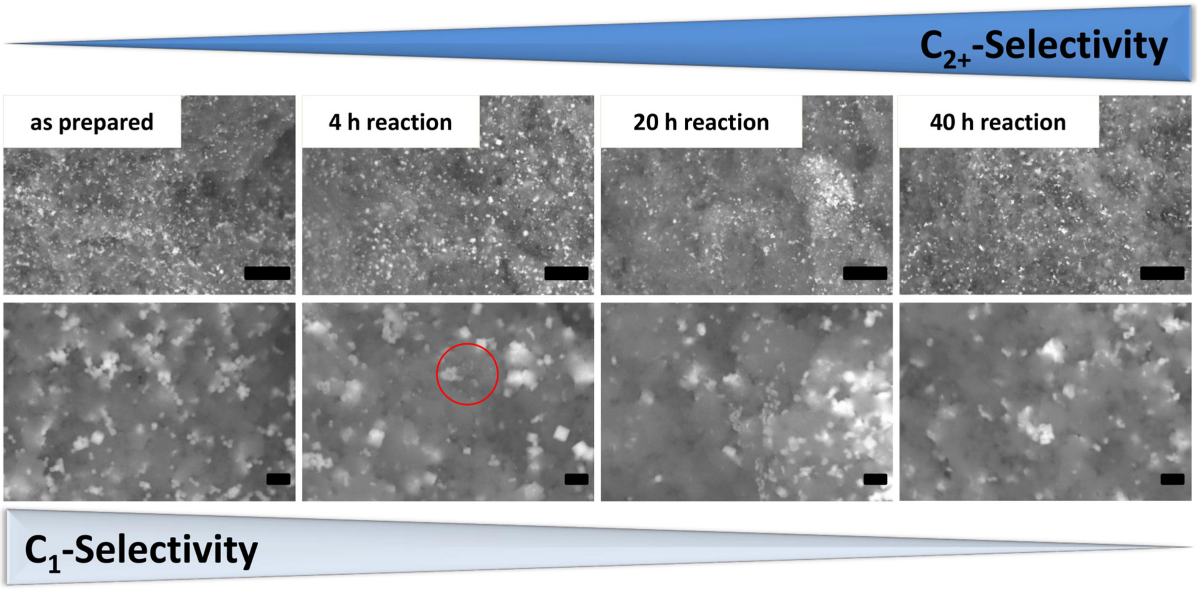

Figure S12. SEM measurements after Flow-Cell testing at -300 mA cm^{-2} on the supported particles. SEM-COMPO images of the supported (23 wt% metal loading) after different times of reduction at constant current of -300 mA cm^{-2} in the Flow-Cell. Scale bars represent 2000 nm in the first row and 200 nm in the second row. The formation of small particle is indicated by a red circle in the image after four hours of reaction time.

Evolution of the Cathode-Potential vs RHE during stability tests in the Flow-Cell.

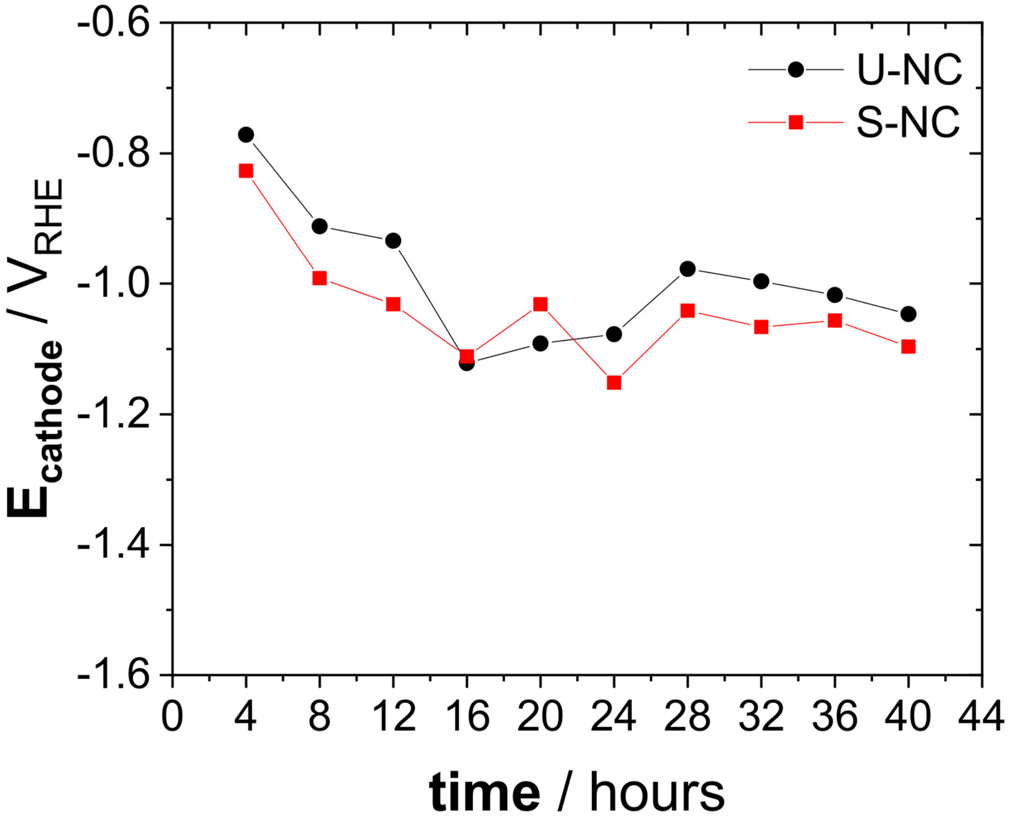

Figure S13. Temporal evolution of potential during stability testing in the Flow-Cell. The cathode potential vs. IR-free RHE as a function of time for the unsupported, U-NC, and supported, S-NC, (23 wt% metal loading) catalyst at -300 mA cm^{-2} over the duration of 40 h. Every 4 h the resistance was determined by a PEIS measurement, which was used to account for the ohmic drop. Lines to guide the eye

Morphological and structural changes of the Cu_2O cubes during CO_2RR.

As our *quasi in situ* XPS results suggest, negative potentials during reaction cause a complete reduction of the near-surface region of the catalyst. This process is fast in the case of the unsupported, as well as the supported nanocubes and progresses towards a completely metallic overlayer within one hour of reaction time. Nevertheless, our *operando* XAS results show that the unsupported nanocubes are not fully reduced during reaction and can retain a significant fraction of 20 % Cu(I) in the sub-surface structure for extended periods of time. Additionally, during this process a defect-rich structure is formed displayed by a clearly lower CN than that expected of bulk Cu, based on our analysis of EXAFS data.

The nanocubes are generally morphologically unstable under those conditions and start to merge with adjacent NPs to form larger intertwined structures. Here, the dispersion on the carbon support shows a distinct effect for the morphological evolution, allowing for a structural break-up into smaller Cu clusters compared to the initial particle size. This strong difference in behavior is expected to be caused by the isolation of particles on the support, which decreases the probability for immediate formation of aggregates during the "reduction-driven" morphological break-up. At longer reaction times, however, the dispersed Cu-seeds start to sinter to minimize surface energy, facilitated by their high mobility on the weak-interacting carbon support, and, in turn, their performance aligns to the unsupported system.

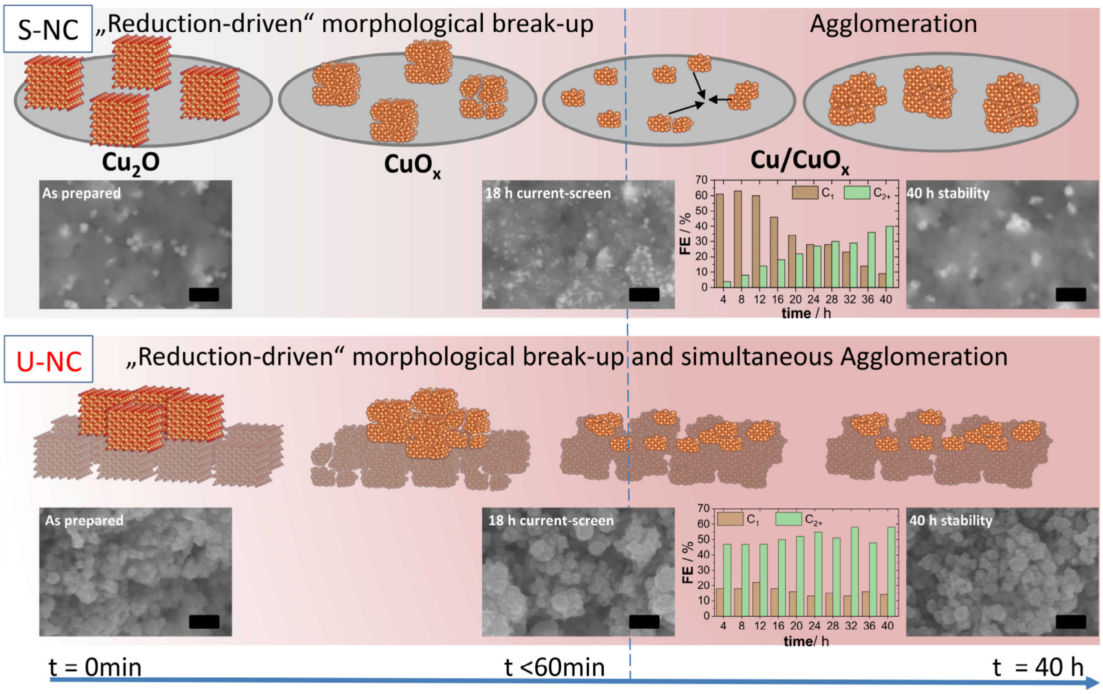

Figure S14. Schematic representation of the morphological and structural changes of the shaped cubic Cu_2O during CO_2RR in a flow-electrolyzer*. Schematic representation of the morphological and structural degradation of the supported Cu nanocubes (23 wt%), S-NC, and unsupported Cu nanocubes, U-NC, during CO_2RR in a flow-electrolyzer. Top-view SEM images of the gas diffusion electrode as prepared, after 18 h of selectivity screen and after 40 h of stability test at -300 mA cm^{-2}, as well as the temporal evolution of the C_{2+} and C_1 Faradaic efficiency during 40 h of stability test at -300 mA cm^{-2} are displayed for U-NC and S-NC, respectively.*

References

[1] X.-W. Liu, F.-Y. Wang, F. Zhen, J.-R. Huang, *RSC Adv.* **2012**, *2*, 7647-7651.

[2] N. Leonard, W. Ju, I. Sinev, J. Steinberg, F. Luo, A. S. Varela, B. Roldan Cuenya, P. Strasser, *Chem. Sci.* **2018**, *9*, 5064-5073.

[3] M. C. Biesinger, L. W. M. Lau, A. R. Gerson, R. S. C. Smart, *Appl. Surf. Sci.* **2010**, *257*, 887-898.

[4] a) A. Łukomska, J. Sobkowski, *J. Electroanal. Chem.* **2004**, *567*, 95-102; b) G. M. Brisard, E. Zenati, H. A. Gasteiger, N. Markovic, P. N. Ross, *Langmuir* **1995**, *11*, 2221-2230.
